# Patterns of genomic differentiation between two Lake Victoria cichlid species, *Haplochromis pyrrhocephalus* and *H.* sp. ‘macula’

**DOI:** 10.1186/s12862-019-1387-2

**Published:** 2019-03-04

**Authors:** Shohei Takuno, Ryutaro Miyagi, Jun-ichi Onami, Shiho Takahashi-Kariyazono, Akie Sato, Herbert Tichy, Masato Nikaido, Mitsuto Aibara, Shinji Mizoiri, Hillary D. J. Mrosso, Semvua I. Mzighani, Norihiro Okada, Yohey Terai

**Affiliations:** 1 Department of Evolutionary Studies of Biosystems, SOKENDAI (The Graduate University for Advanced Studies), Shonan Village, Hayama, Kanagawa, 240-0193 Japan; 2 Graduate School of Bioscience and Biotechnology, Tokyo Institute of Technology, 4259 Nagatsuta-cho, Midori-ku, Yokohama, 226-8501 Japan; 3 Department of Biological sciences, Tokyo Metropolitan University, 1-1 Minamiosawa, Hachioji, Tokyo, 197-0397 Japan; 4 JST (Japan Science and Technology Agency), NBDC (National Bioscience Database Center), 5-3, Yonbancho, Chiyoda-ku, Tokyo, 102-0081 Japan; 5 Department of Anatomy and Cytohistology, School of Dental Medicine, Tsurumi University, 2-1-3 Tsurumi, Tsurumi-ku, Yokohama, 230-8501 Japan; 6 Max-Planck-Institut für Biologie, Abteilung Immungenetik, Corrensstrasse 42, D-72076 Tübingen, Germany; 7 School of Life Science and Technology, Department of Life Science and Technology, Tokyo Institute of Technology (Tokyo Tech), 2-12-1, Ookayama, Meguro ward, Tokyo, Japan; 8 Tanzania Fisheries Research Institute (TAFIRI), Mwanza, Tanzania; 9 Department of Life Sciences, National Cheng Kung University, 701 Tainan, Taiwan; 10 Foundation for Advancement of International Science (FAIS), Tsukuba, Japan

**Keywords:** Cichlids, Population genomics, Adaptation, Speciation, Genomic islands of speciation

## Abstract

**Background:**

The molecular basis of the incipient stage of speciation is still poorly understood. Cichlid fish species in Lake Victoria are a prime example of recent speciation events and a suitable system to study the adaptation and reproductive isolation of species.

**Results:**

Here, we report the pattern of genomic differentiation between two Lake Victoria cichlid species collected in sympatry, *Haplochromis pyrrhocephalus* and *H.* sp. ‘macula,’ based on the pooled genome sequences of 20 individuals of each species. Despite their ecological differences, population genomics analyses demonstrate that the two species are very close to a single panmictic population due to extensive gene flow. However, we identified 21 highly differentiated short genomic regions with fixed nucleotide differences. At least 15 of these regions contained genes with predicted roles in adaptation and reproductive isolation, such as visual adaptation, circadian clock, developmental processes, adaptation to hypoxia, and sexual selection. The nonsynonymous fixed differences in one of these genes, *LWS*, were reported as substitutions causing shift in absorption spectra of LWS pigments. Fixed differences were found in the promoter regions of four other differentially expressed genes, indicating that these substitutions may alter gene expression levels.

**Conclusions:**

These diverged short genomic regions may have contributed to the differentiation of two ecologically different species. Moreover, the origins of adaptive variants within the differentiated regions predate the geological formation of Lake Victoria; thus Lake Victoria cichlid species diversified via selection on standing genetic variation.

**Electronic supplementary material:**

The online version of this article (10.1186/s12862-019-1387-2) contains supplementary material, which is available to authorized users.

## Background

The molecular basis of the incipient stage of speciation is of great interest in genetics, ecology, and evolutionary biology [1]. Cichlid species in Lake Victoria are a suitable model system to study this stage of speciation [2]. Lake Victoria harbors more than 500 endemic cichlid species [3, 4]. They are thought to have experienced an explosive adaptive radiation during a very short evolutionary period because Lake Victoria dried up at the end of the Pleistocene and was refilled only 15,000 years ago [5, 6]. Indeed, levels of genetic differentiation among species are low, and the species share a large number of nucleotide polymorphisms including differentiated variants shown by studies using a small number of genetic markers, restriction site associated DNA (RAD) data, and whole genome sequencing data [7–14]. Nevertheless, fixed genetic differences between species are expected at loci responsible for adaptive traits and, as a consequence, for speciation. One of the best examples is the long wavelength-sensitive opsin gene (*LWS*), which exhibits a high level of genetic differentiation with fixed genetic differences among Lake Victoria cichlid species [15–19]. As expected, *LWS* alleles are variable among species adapted to different light environments created by different turbidities and different depths [19] and are responsible for speciation by sensory drive [15, 17]. Such variation of *LWS* alleles among species would have originated from the admixture of two divergent lineages [20]. The other gene with fixed genetic differences is the rod opsin gene (*RH1*) for scotopic vision. *RH1* alleles are differentiated among species from different turbidities and depths, and adapted to their ambient light environments [19].

The joint effect of gene flow and divergent selection shapes the pattern of genomic differentiation between an incipient species pair. At the very beginning of this stage, a small part of genes could be involved in reproductive isolation and/or local adaptation [21]. In the latter case, divergent selection acts on these genes, where one allele is advantageous in a species and the other allele is advantageous in its counterpart because separated populations adapt to different niches/environments. Gene flow actually occurs around the target sites of divergent selection but offspring of migrants with the non-adaptive allele are immediately selected out from the species, and as a consequence, the effective migration rate is decreased. On the other hand, gene flow is allowed in other genomic regions and suppresses differentiation, leading to the heterogeneity of genetic differentiation [21–24].

Indeed, the lines of empirical evidence of speciation with gene flow have been recently increased, in plants [25], insects [26, 27], and cichlid species [7, 8, 28, 29]. Despite gene flow, highly differentiated genomic regions between species exist in the genome, and these regions bear genes related to local adaptation such as pigmentation and visual perception in crows [30], beak shape in Darwin’s finches [31], and *RH1* for scotopic vision in cichlids [28]. Also, fixed nucleotide differences have been observed in such short genomic regions that emerge only when divergent selection effectively acts [28, 30–34].

Recently, this pattern of genomic differentiation has been reported between two Lake Victoria species, *Pundamillia nyererei* and *P. pundamillia* that both live in rocky habitats [7, 8]. On the other hand, many Lake Victoria cichlid species are distributed along the bottom where there is a soft sandy–muddy substrate, and the pattern of genomic differentiation between species from a sandy–muddy bottom has not been analyzed. The differentiated genomic regions between closely related species from a sandy–muddy bottom may contain candidate genes related to adaptation to micro-habitats, and these candidate genes provide an opportunity to study the incipient evolutionary process in a soft-bottom, benthic ecosystem.

**Fig. 1:**
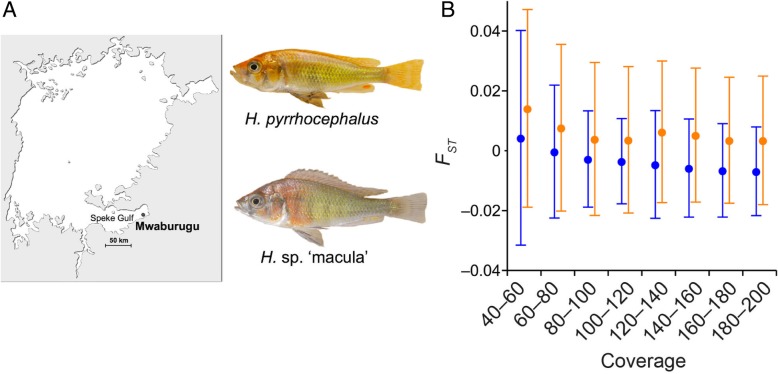
(**a**) Adult males of *Haplochromis pyrrhocephalus* and *H*. sp. ‘macula’ with nuptial coloration. A sampling location from the Mwaburugu region is shown by a dot on the map of Lake Victoria. (**b**) Average *F*_ST_ values (±1 SD) against coverage for Pool-seq data. The blue and orange dots represent observed and simulated values under panmixia

In this study, we focused on two cichlid species from Mwaburugu to reveal the pattern of genomic differentiation between closely related species living in a sandy–muddy habitat. Mwaburugu is a consistently shallow area (2–3 m) with a sandy–muddy bottom, located in the eastern region of Speke Gulf (Fig. 1a). The two species exhibit morphological and behavioral differences (Fig. 1a). *H. pyrrhocephalus* inhabits the middle layer (mainly 7–13 m in Mwanza gulf, south part of Lake Victoria) [35], and *H*. sp. ‘macula’ is a demersal species [36, 37]. In Mwaburugu, however, the two species distribute in sympatry at a 1- to 3-m depth (Fig. 1a). *H*. sp. ‘macula’ is a phytoplankton eater, while *H. pyrrhocephalus* is a zooplanktivore [38]. Males of these two species exhibit different nuptial colorations [3], but the distribution of the hue index values largely overlap and are different from other species in Mwaburugu [18]. In cichlids, color perception is important for mate choice [17, 39–41]. Therefore, we expected that the reproductive isolation of these two species may be incomplete, and as a consequence, the genomic differentiation between them may be low. Indeed, we found that most of their genome did not show significant differentiation between the two species.

## Results

### Summary of population genetic statistics

We performed population genomic analyses in *H. pyrrhocephalus* and *H*. sp. ‘macula’ from the same locality in Lake Victoria (Fig. 1a). We sampled 20 individuals (10 males and 10 females) for each species, extracted DNA, and constructed Pool-seq libraries (Methods). After mapping the paired-end short reads to the reference genome sequence, the final coverage was ~ 34× in both species. We extracted 4,967,102 and 5,109,870 sites with 80–200× coverage for *H. pyrrhocephalus* and *H*. sp. ‘macula,’ respectively (Additional file 2: Text S1). We first inferred the site frequency spectrum (SFS) for each species [42]. We estimated 329,613 (6.64%) and 322,956 (6.32%) polymorphic sites in *H. pyrrhocephalus* and *H*. sp. ‘macula,’ respectively. The folded SFSs were very similar between the two species (Additional file 1: Figure S1A), and almost 70% of segregating sites were inferred to be singletons in each species. Nucleotide diversity [43] was 0.00717 and 0.00678 for the two species. Tajima’s *D* values [44] were highly negative (less than − 2 in both species), indicating an excess of rare alleles in each species. This observation suggests population expansion [45] accordingly, and we inferred demographic parameters based on the SFSs using δa/δi [46] (Additional file 2: Text S1). As expected, we detected rapid population expansion after the colonization of Lake Victoria; the current effective population size was on the order of 10^6^ (Additional file 2: Text S1).

Note that the power and accuracy of a Pool-seq analysis would be low for allele frequency estimation, especially for alleles at lower frequency [47]. Nevertheless, nucleotide diversity and Tajima’s *D* values are consistent with previous estimates based on the Sanger method [12, 48]. We further note that we utilized sites with a much higher coverage than the genomic average, and high coverage can indicate repetitive regions. After excluding sites within repetitive regions, we obtained nearly the same statistics for each species (Additional file 2: Text S1).

### Two Lake Victoria cichlid species would be close to a single panmictic population

We measured the level of population differentiation between the two species. We estimated the allele frequency at every site given the estimated rate of sequence errors (Materials and Methods, Additional file 2: Text S1). We used 7,516,409 polymorphic sites, at which the coverage was ≥40 for both species. When the minor allele frequency in the total dataset was higher than 0.05, > 80% of sites exhibited shared polymorphisms, as observed in Lake Victoria cichlids [7–14, 48], suggesting that the species are closely related. We applied *F*_ST_statistics [49], which is suitable to quantitatively assess the level of differentiation between such closely related species or populations. This statistic is calculated by.

1 $$ {F}_{ST}=1-\frac{2{T}_W\mu }{2{T}_B\mu }=1-\frac{\pi_W}{\pi_B} $$

where *T*_W_ and *T*_B_ represent the coalescent time of samples from the same population and that of samples from different populations, respectively; μis the mutation rate per generation, and π_W_ and π_B_ represent the average nucleotide diversity within each population and the average pairwise nucleotide divergence between the two populations, respectively. To measure the level of population differentiation, *T*_W_ was used as a control. When two sets of samples are from the same population, *T*_W_ and *T*_B_ are expected to be equal and *F*_ST_ is close to 0.

We calculated *F*_ST_ for each of the 7,516,409 segregating sites. Because the accuracy of allele frequency estimates is low when coverage is low [47], *F*_ST_ is expected to become relatively high at such sites. As expected, we found a slight, but detectable, negative correlation between *F*_ST_ and coverage (blue plot in Fig. 1b). Nevertheless, average *F*_ST_ values were around zero even in alleles with low coverage (< 0.0043; Fig. 1b), indicating almost no population differentiation.

**Fig. 2:**
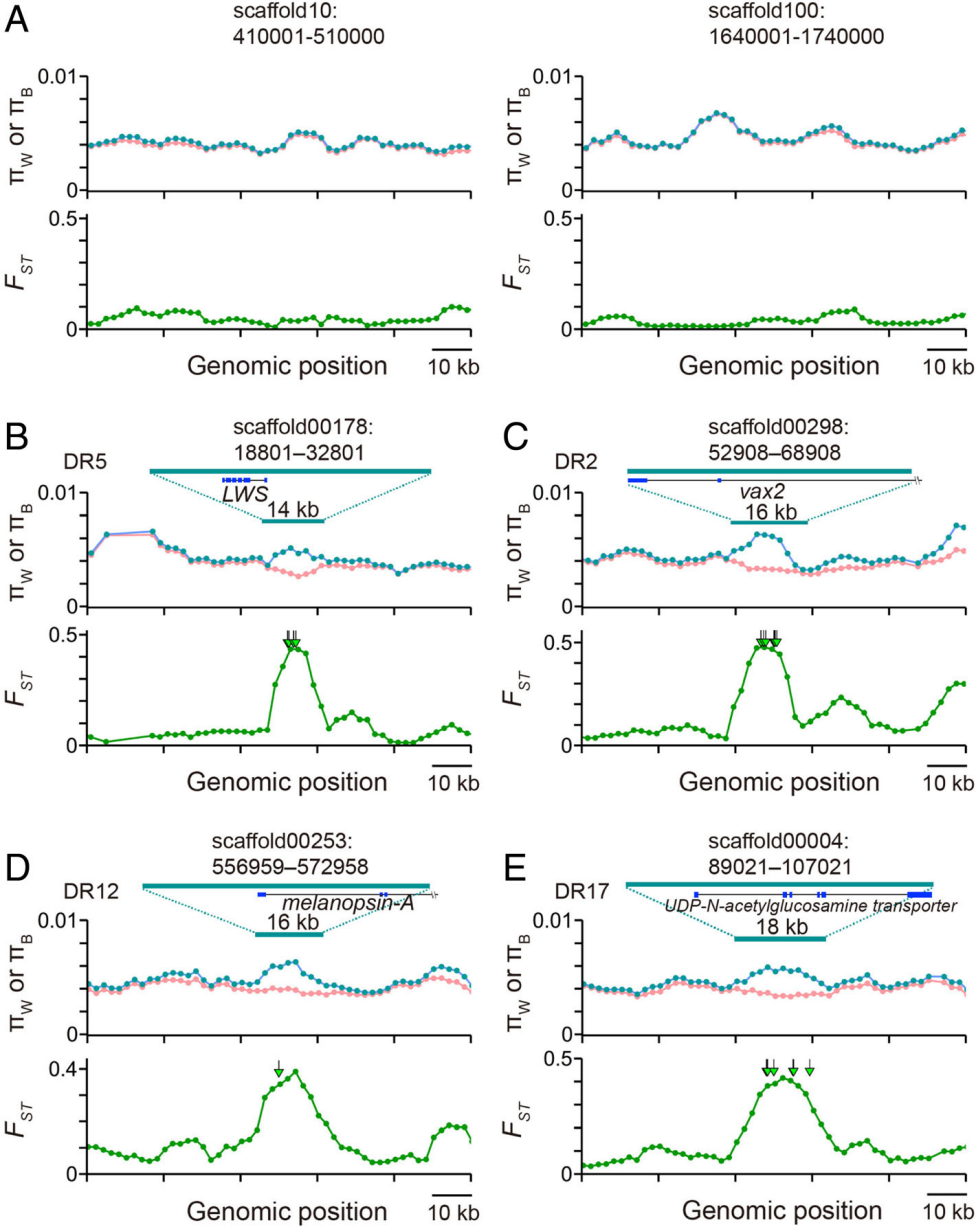
The spatial patterns of the average values of intraspecies nucleotide diversity in *H. pyrrhocephalus* and in *H*. sp. ‘macula’ (π_W_; pink); average pairwise nucleotide divergence between the species (π_B_; blue); and *F*_ST_ (green). (**a**) Typical genomic regions. (**b**–**e**) Candidates of the target genes of divergent selection. The green arrows represent fixed nucleotide differences between species. Green and blue solid lines indicate differentiated regions (DRs) and exons of genes, respectively. The positions of DRs are described on the right side of the green lines

We examined whether we could treat the two species as a single panmictic population. In theory, when the migration rate is high enough (i.e., when the population migration rate, 4 *Nm*, is higher than 10, where *N* is the effective population size and *m* is the migration rate per gamete per generation), the pattern of nucleotide polymorphisms in samples from two populations is expected to be similar to that in samples from a single population [50]. To test this, a standard coalescent simulation is not sufficient because we used Pool-seq data. Thus, to generate null distributions of *F*_ST_ values given our coverage, we simulated both the coalescent process under panmixia and the Pool-seq process with the inferred population expansion (Additional file 2: Text S1). The distributions of simulated *F*_ST_ values exhibited a similar tendency (orange plot in Fig. 1b), though the observed distributions were slightly skewed toward negative values. When a single segregating site was analyzed, *F*_ST_ statistics became negative when allele frequencies in the two sample sets were very similar. This indicates a slight excess of the proportion of segregating sites with very similar allele frequencies between the two species. The cause of this excess is not known, but the observed and expected distributions were largely overlapping. Thus, we reasoned that the two species might be close to a single panmictic population (see also Fig. 2a).

We have three caveats in this section. First, as in the previous section, we excluded sites in repetitive regions, and we obtained essentially the same results as in Fig. 1b (Additional file 2: Text S1). The second caveat is that a Pool-seq analysis is less powerful for detecting population differentiation, especially when minor allele frequency is low [47]. However, in our case we could detect population differentiation when *F*_ST_ was ≥0.01, and when the sample size was set to be 40 in each population (Additional file 2: Text S1). Furthermore, we excluded singletons and obtained essentially the same results as in Fig. 1b. Finally, the females of *H*. sp. ‘macula’ are morphologically similar to other *Haplochromis* or *Enterochromis* species*,* and it is possible that these female specimens included misidentification of species. To avoid the misclassification of species, we performed the same analysis only in male individuals. Despite the reduction in sample size, we obtained essentially same result (Additional file 3: Figure S2).

### Identification of highly differentiated genomic regions

Despite minimal genomic differentiation, *H. pyrrhocephalus* and *H*. sp. ‘macula’ exhibit differences in morphology and habitat [3]. Thus, we performed a genome scan to search for candidate genes that are subject to divergent selection using sites with 20–200× coverage in both species (Additional file 2: Text S1). We used 10-kb window size with 2-kb increments for the scan, and calculated π_W_, π_B_, and *F*_ST_ for each window. The spatial distributions of π_W_ and π_B_ are almost the same across the genome, indicating that *T*_W_ and *T*_B_ are almost the same and *F*_ST_ values are close to zero (see two typical genomic regions in Fig. 2a) due to the panmixia of the two species (Fig. 1b). On the other hand, the *LWS* gene, a prime example of target genes under divergent selection [15, 17, 18], exhibited a remarkable pattern. The spatial patterns of π_W_, π_B_, and *F*_ST_ around *LWS* are shown in Fig. 2b. We observed that π_B_ values are significantly higher than π_W_, and as a consequence, there is a clear peak of *F*_ST_ in the 14-kb region that includes the *LWS* gene. Despite the low accuracy of Pool-seq, these statistics are very close to the estimates reported in a previous study, which determined the sequences by the Sanger method [18]. Outside the 14-kb region, π_W_ and π_B_ values are almost equal as in Fig. 2a. Furthermore, we found seven fixed nucleotide differences between *H. pyrrhocephalus* and *H*. sp. ‘macula’ within the peak (green triangles in Fig. 2b) that indicate strong signatures of divergent selection under the pressure of extensive migration.

To screen candidate genes under divergent selection, we initially filtered windows with the top 0.1% of *F*_ST_ values (*F*_ST_ > 0.372). We performed a neutrality test to see if such high *F*_ST_ values are observed without natural selection. We simulated a coalescent process and Pool-seq process with 20× coverage to maximize the variance of a null distribution (Additional file 2: Text S1), and obtained a very small *P*-value (*P* < 10^− 5^; false discovery rate < 0.014). We further screened such windows that exhibited a clear peak of *F*_ST_ values as in *LWS* genes (Fig. 2b) and fixed nucleotide differences within the peaks. In total, we detected 21 highly differentiated regions (14–28 kb). Hereafter, we focused on these 21 short differentiated regions (DRs), and three examples of DRs are shown in Fig. 2c-e and the others in Additional file 4: Figure S3. We searched for genes in the DRs by BLASTN and explored their biological roles in terms of adaptation and speciation. Nineteen out of 21 DRs included 1–3 genes (Table 1), and 28 total genes were found in the DRs.

**Table 1:** Genes in DRs

DRs	Gene name	Predicted functions related to adaptation and speciation ^c^
DR1^a^	diaphanous	Developmental process, cell movement, auditory
DR2^a^	ventral anterior homeobox 2	Development of retina
DR3^a^	prostaglandin d2 receptor 2	Unknown
	G-protein coupled receptor 4 (GPR4)	Adaptive to different oxygen concentration
	UDP-glucuronosyltransferase 2b15	Unknown
DR4	hemicentin-1	Unknown
DR5^a^	long wavelength-sensitive opsin (LWS)	Speciation by sensory drive
DR6^a^	netrin receptor UNC5c	Brain development
DR7	general transcription factor IIH subunit 1	Unknown
DR8^a^	intestinal mucin	Host-specific microbiota composition
DR9^a^	hepatocyte growth factor receptor	Morphogenesis for the muscles of fins, affecting mobility
DR10^a^	*tbx3*^b^ (30 kb downstream from DR10)	Developmental process
DR11^a^	ap-4 complex subunit epsilon	Unknown
	cytochrome p450 aromatase type II	Sexual differentiation of the brain and reproductive behavior
	gliomedin	Unknown
DR12^a^	melanopsin A	Photic regulation of circadian clocks
DR13	No gene	–
DR14	Uncharacterized protein	–
	Uncharacterized protein	–
DR15	Uncharacterized ncRNA	–
DR16^a^	aryl hydrocarbon receptor nuclear translocator	Adaptive to different oxygen concentrations
DR17	UDP-n-acetylglucosamine transporter	Unknown
	U3 small nucleolar ribonucleoprotein protein imp3	Unknown
DR18^a^	peptidyl-prolyl cis-trans isomerase H	Unknown
	transcription initiation factor TFIID subunit 10	Early embryonic development
	G-protein coupled receptor 160	Unknown
DR19^a^	type II cytoskeletal 5	Epidermis development
DR20^a^	hydroperoxide isomerase aloxe3	Epidermis development
	macrophage mannose receptor 1	Unknown
DR21^a^	Ras-related protein rab-11a	Unknown
	RNA-binding protein mex3a	Brain aging

^a^DRs contained genes with predicted roles for adaptation and speciation

^b^This DR did not contain a gene, but *tbx3* was located 30 kbp downstream from this DR

^c^References are listed in Additional file 6: Table S1

As mentioned above, allele frequency estimates in regions of low coverage are not accurate, especially for alleles with low frequencies [47]. Thus, we repeated the analysis, discarding segregating sites with minor allele frequency ≤ 0.05, and identified the same set of 21 regions. Furthermore, we verified the fixed nucleotide differences by Sanger sequencing for 5 of 21 loci (we selected a subset of 5 DRs due to limited amounts of DNA samples) (Additional file 1: Figure S1C). To avoid the misclassification of species, we repeated the analysis using only male samples as in the previous section. We obtained essentially the same result (Additional file 3: Figure S2B) as in the *LWS* genes (Fig. 2b) and in other DRs.

### The pattern of polymorphisms around the target site of divergent selection

The hitchhiking effect of divergent selection under the pressure of migration is more limited than that of positive selection (i.e., selective sweep). To show this, we performed a population genetic simulation under a simplified model (Methods). We assumed an isolation with migration model with two populations (populations 1 and 2), into which we incorporated mutation, recombination, migration, divergent selection, and random genetic drift. The locus I is subject to divergent selection, and has two alleles, *A* and *a*. The *A* allele is advantageous in population 1, and the *a* allele is advantageous in population 2. At the start of simulations, locus I is monomorphic for the *A* allele, and a linked neutral locus (locus II) is under mutation-drift equilibrium. At the time *T* = 0, we introduced the *a* allele in population 2 with the initial frequency of 1/2 *N* and ran simulations for 8 *N* generations. The beneficial alleles (allele *A* in population 1 and allele *a* in population 2) are expected to immediately reach an equilibrium frequency, $\widehat{p}$, when population size is infinite:

2 $$ \widehat{p}=-\frac{m}{s}+\frac{1}{2}+\sqrt{{\left(\frac{m}{s}\right)}^2+\frac{1}{4}} $$

where *m* is migration rate per generation, and *s* is the selection coefficient. As long as *s* is much higher than *m* (e.g., *s*/*m* > > 5), $\widehat{p}$ is very close to 1. We ran simulations 100,000 times each with a variety of pairs of *m* and *s*, and calculated expected π_W_, π_B_, and *F*_ST_ at several time points. We confirmed that the frequencies of the beneficial alleles in both populations quickly reached $\widehat{p}$ in finite populations, and the patterns of polymorphisms were qualitatively consistent among the pairs of *m* and *s*.

**Fig. 3:**
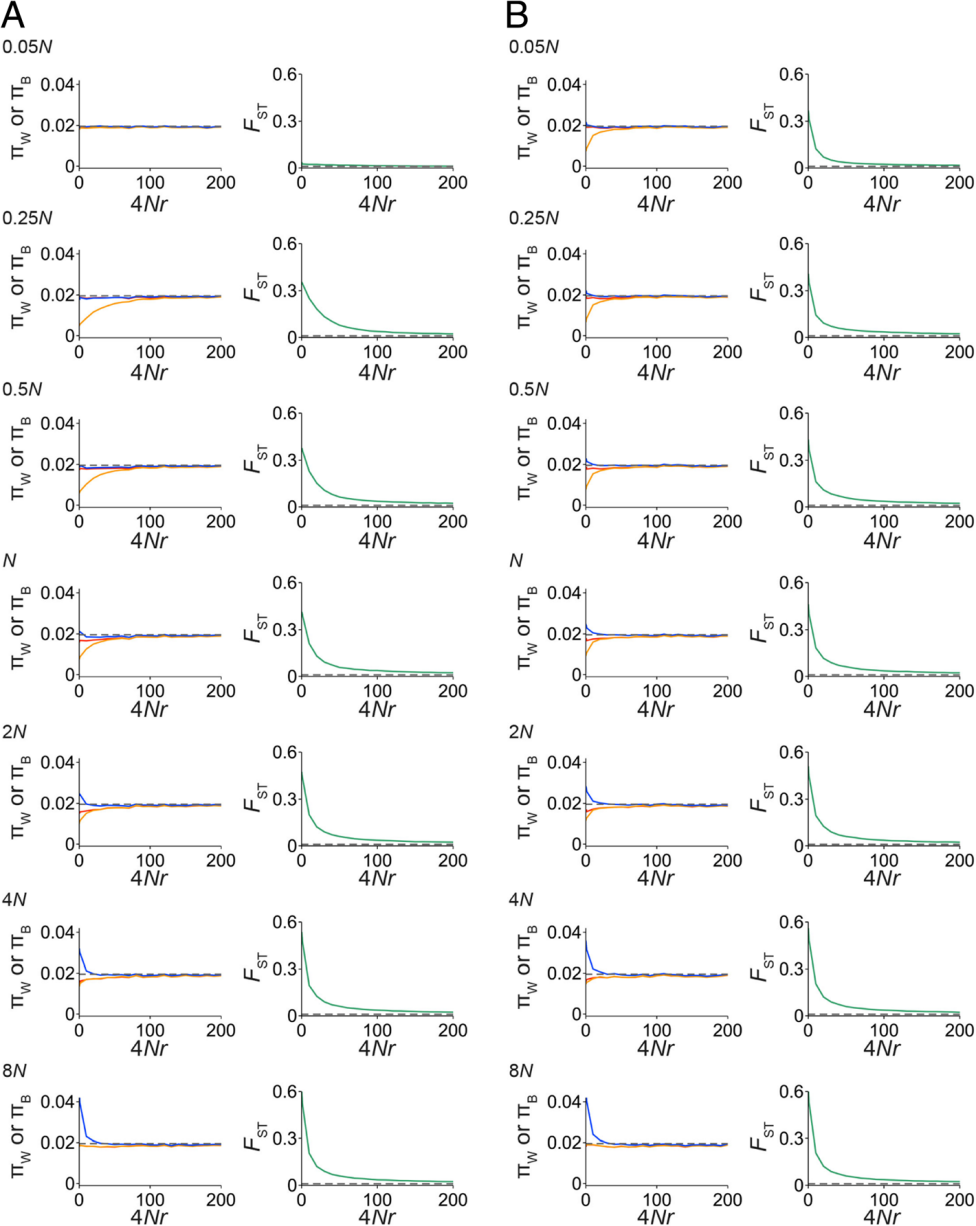
The hitchhiking effect of divergent selection when a new beneficial allele arises by a new mutation (**a**) and is derived from standing variation (**b**). The *x*-axes represent distance from the target site of divergent selection (scaled by the population recombination rate), and the *y*-axes represent nucleotide diversity in population 1 (π_W_; red), π_W_ in population 2 (orange), average pairwise nucleotide divergence between species (π_B_; blue), and *F*_ST_ (green). Dashed gray lines indicate the theoretical expectations of π_W_ and *F*_ST_ under neutrality

We show the result with 4 *Nm* = 50, and 4*Ns* = 400 in greater detail in Fig. 3a. First, the *a* allele quickly reaches $\widehat{p}$ in population 2 which causes a strong reduction of π_W_ in long genomic regions such as in the case of a selective sweep event [51, 52]. However, the signature of selective sweep is quickly eliminated by the effects of migration and recombination, and a short differentiated region appears with fixed nucleotide differences as shown in Fig. 2b-e and Additional file 3: Figure S3. This situation is very similar to the process of neofunctionalization of duplicated genes (an analog to divergent selection in our model) under the pressure of interlocus gene conversion (analogs to migration and recombination), where a short differentiated peak between duplicates appears around the target site of neofunctionalization [53]. We further simulated the situation in which the *a* allele is derived from standing genetic variation because it has been proposed that adaptive variants are derived from standing variation ([20]; see the section “Lake Victoria cichlid species diversified via selection on standing genetic variation” below). We ran simulations with *s* = 0 until the frequency of the *a* allele reached 20%, and then divergent selection started to act. The result is shown in Fig. 3b, and we found that the shrinkage of the differentiated region is faster than that in Fig. 3a.

A high false positive rate for the *F*_ST_-outlier approach has been pointed out previously, and using absolute nucleotide divergence (or π_B_) is recommended [54, 55]. *F*_ST_ can be increased without divergent selection when π_W_ is reduced as in Eq. (1), for example, by a classical selective sweep or by background selection. When one focuses on highly differentiated species, we may not be able to ignore this flaw of the *F*_ST_-outlier approach (i.e., when *T*_B_ is much older than *T*_W_; *F*_ST_ > 0.1 as in [54]). However, a high false positive rate is expected when using π_B_ in low-differentiated, young species pairs with extensive migration such as *Haplochromis* species (Figs. 1b and 2). This is because high π_B_ regions occasionally appear due to the large variance of coalescent time in the ancestral population. If such regions have evolved in a neutral manner, π_W_ values are expected to be equal to π_B_ due to migration, and *F*_ST_ is close to zero. Thus, employing *F*_ST_ should perform better in our case.

In our DRs, the possibility of a classical selective sweep without divergent selection is unlikely. As shown in Fig. 3, we observe the sweep-like strong reduction of π_W_ that actually increases *F*_ST_. However, without divergent selection, such reduction of π_W_ is quickly eliminated. Both divergent selection and migration are required to maintain short differentiated regions as observed in Fig. 2b-e and Additional file 3: Figure S3. Background selection is also unlikely. The effect of background selection is remarkable on regions with low recombination rates. Purifying selection purges deleterious mutations, and also linked neutral variants together. As a consequence, π_W_ is slightly reduced in relatively long genomic regions [56], and *F*_ST_ values become high. However, the lengths of DRs are very short (14–28 kb; Fig. 2b–e; Additional file 3: Figure S3). Furthermore, the inferred population sizes in *Haplochromis* species are fairly large (Additional file 2: Text S1), and therefore the population recombination rate (4*Nr*, where *N* is effective population size and *r* is recombination rate per generation) is so high that recombination effectively reduces the effect of background selection. More importantly, in both cases, we would not expect to observe fixed nucleotide differences under the pressure of migration without divergent selection, however, we did (Fig. 2b-e, Additional file 3: Figure S3).

### The roles of fixed differences in DRs

Among the genes in DRs (Table 1), six nonsynonymous fixed differences were located in the coding region of *LWS* (Additional file 1: Figure S1C). These nonsynonymous substitutions make 8 and 9 nm shifts in the absorption spectra of LWS photo-pigments, with 11-*cis* retinal (A1-) and 11-*cis* 3- dehydroretinal (A2-derived retinal), respectively [18], suggesting that the fixed differences are responsible for the functional difference of LWS pigments between two species.

**Fig. 4:**
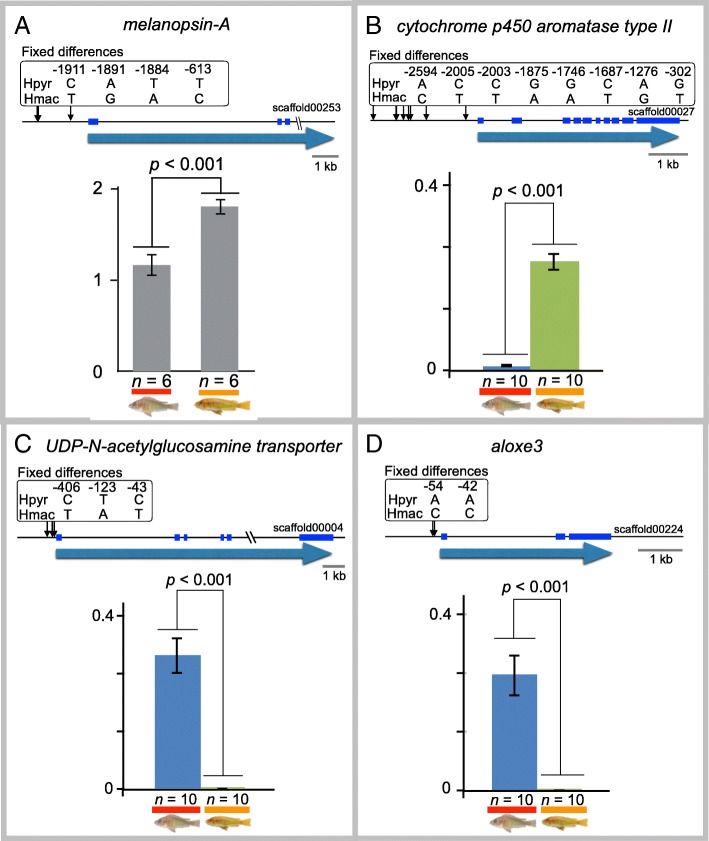
(**a**–**d**) Different expression levels of genes between *H. pyrrhocephalus* (Hpyr) and *H*. sp. ‘macula’ (Hmac). Expression was analyzed by qPCR using RNA from eyes (**a**, each of three individuals) and the anterior part of lateral skin (**b**–**d**, 10 individuals each). Gene names are shown at the top of the panels. The direction of blue arrows indicates the gene direction. Black arrows and blue solid lines represent the positions of fixed differences and exons of genes, respectively. The numbers and nucleotides under the numbers in the rectangles indicate the position from the initiation codon and fixed nucleotides in *H. pyrrhocephalus* (upper) and *H*. sp. ‘macula’ (lower), respectively

In contrast, four fixed differences in another opsin, *melanopsin A*, were not located in the coding region, but are in the upstream region of the gene (Fig. 4a), raising the possibility of differential expression. We quantified the expression level of this gene in the eyes of six individuals each from *H. pyrrhocephalus* and *H*. sp. ‘macula’ by real-time qPCR. We detected a significant difference in expression between species (Fig. 4a), indicating the possibility that these substitutions may cause the expression difference of *melanopsin A* in these two species.

The color pattern of cichlids is one of the most variable traits among species in Lake Victoria; therefore, we performed a pooled RNA-seq analysis to screen for the candidates of differentially expressed genes in the anterior part of the lateral skin of these two species. In total, 172 contigs showed differential expression between the two species (*P* < 0.05). These included three genes in DRs, *P450 aromatase*, *UDP-N-acetylglucosamine transporter*, and *aloxe3*. The fixed differences in these genes were also located in their upstream regions (Fig. 4b–d). Therefore, we quantified the expression levels of these genes in the anterior part of the lateral skin of ten individuals each from both species. In all three genes, the expression levels in the anterior part of the lateral skin were completely different between the two species (Fig. 4b–d, *P* < 0.001). These results also indicate the possibility of differential expression of genes caused by the fixed differences.

### Lake Victoria cichlid species diversified via selection on standing genetic variation

**Fig. 5:**
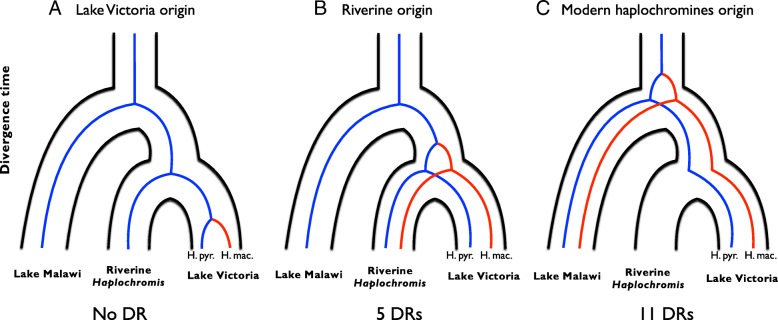
The origins of mutations in DRs. Three genealogies represent the accumulation of mutations tracing back to the common ancestral species of (**a**) Lake Victoria species, (**b**) Lake Victoria and riverine *Haplochromis* species, and (**c**) Modern haplochromines. Blue and red lines indicate *H. pyrrhocephalus* and *H*. sp. ‘macula’ alleles, respectively

We inferred the origin of the putative adaptive variants (i.e., fixed differences) in cichlid species. We determined the sequences (~ 1 kbp), including the fixed differences, within 16 DRs from Lakes Victoria, Malawi, Tanganyika, and riverine *haprochromis* species. We constructed phylogenetic trees based on our sequences and orthologous sequences in other cichlid genomes [14]. No tree for any DR showed monophyly of Lake Victoria species (Fig. 5a and Additional file 5: Figure S4A), suggesting that these alleles in DRs arose as presumably neutral variants in the ancestral population of the two species. By contrast, five trees showed monophyly of the Lake Victoria superflock, including species from Lake Victoria and surrounding rivers [9, 16] (Additional file 5: Figure S4F), suggesting that the adaptive variants arose in the common ancestor of this monophyletic clade (riverine origin: Fig. 5b and Additional file 5: Figure S4B). The origin of variants in these five DRs are coincident with the scenario that two divergent lineages admixed in the ancestor of the Lake Victoria superflock [20]. *Astatotilapia burtoni* and species from Lake Victoria, surrounding rivers, Lake Malawi and tribe Tropheini (Lake Tanganyika) were mixed in a monophyletic clade in the other 11 trees (Additional file 4: Figure S4E), suggesting that the mutations accumulated in the common ancestor of this lineage [Fig. 5c and Additional file 4: Figure S4C, modern haplochromines origin [57]]. Hence, the origin of the adaptive variants can be traced back to some point in the ancestral lineage of modern haplochromines.

## Discussion

In this study, we focused on *H. pyrrhocephalus* and *H.* sp. ‘macula,’ which are distributed in sympatry in Mwaburugu (Fig. 1a) where the bottom is a sandy–muddy substrate with no rocks, woody debris, or other structures.

In the genome scan, we detected 21 DRs (14–28 kb) including 28 genes. *LWS*, located in DR5 (Fig. 2b), is involved in adaptation to different light environments [19] and contributes to speciation by sensory drive [15, 17], suggesting that DRs contain genes responsible for cichlid adaptation and reproductive isolation. DR2 and DR12 contain the vision-related genes ventral anterior homeobox 2 (*vax2*) and melanopsin A, respectively (Fig. 2c and d; Table 1). The *vax2* gene is involved in the regulation of retinal development [58] and melanopsin A is involved in the photic regulation of the circadian clock [59]. *H. pyrrhocephalus* and other pelagic zooplanktivorous species (e.g. *H. laparogramma, H. heusinkveldi*) migrate toward the surface in the evening and stay during the night to forage for zooplankton [35]. *H. pyrrhocephalus* has very large double cone photoreceptor cells in their retinas for high sensitivity to light [60]. The genes involved in retinal development and circadian clock regulation may contribute to the specific features of *H. pyrrhocephalus.* Although we did not analyze other pelagic zooplanktivorous species, DR2 and DR12 might be shared among ecologically similar species with *H. pyrrhocephalus.* Other DRs contained several genes involved in developmental processes, such as the development of the brain (DR6), epidermis (DRs 1, 19, and 20), and fin muscles (DR9) (Table 1, references in Additional file 6: Table S1). The importance of brain activity for sociality and reproduction was reported in cichlids [61] . Both the epidermis structure and fin muscles may be related to species-specific characteristics; the epidermis directly interacts with the external environment and fin muscles affect fish mobility. P450 aromatase (DR11) is responsible for estrogen synthesis and plays a regulatory role in sex determination, gametogenesis, central nervous system development, and reproductive behavior [62], which are important traits for sexual selection. Interestingly, Atlantic cod populations are also differentiated at this gene [63]. Chronic hypoxia has been observed in Lake Victoria [64]. Cichlid species adapt to hypoxia by multiple strategies [65]. Aryl hydrocarbon receptor nuclear translocator gene (DR16) is involved in physiological adaptation to hypoxia [64] and the G-protein coupled receptor 4 (DR3) regulates breathing by CO_2_ stimulation [65]. Lake Victoria cichlid species may experience hypoxia in deep water, heavily vegetated shallow shoreline habitats, and dense algal blooming in open water [66]. Since *H. pyrrhocephalus,* except in Mwaburugu, inhabits deeper water (inferred to be hypoxic) than *H.* sp. ‘macula’, these genes may be involved in adaptation to different oxygen concentrations. Intestinal mucin (DR8) functions as a host-specific determinant affecting the gut microbiota composition, which is important for digestion [67]. Intestinal mucin may be involved in the digestion of species-specific food such as phytoplankton and zooplankton in *H*. sp. ‘macula’ and *H. pyrrhocephalus*, respectively. *Mex3a* (DR21) is associated with brain aging in the short-lived fish *Nothobranchius furzeri*, a model of aging studies [68], and may be related to interspecific differences in aging in Lake Victoria cichlids. In total, we detected genes with predicted roles in adaptation and speciation within at least 15 out of 21 DRs (Table 1), suggesting that the DRs contain genes responsible for adaptation and reproductive isolation. Two DRs (DR10 and DR13) did not contain genes, but might contain regulatory regions of genes (e.g., DR10 was located 30 kb upstream of *tbx3*).

How are the observed fixed differences related to functional differences in genes in DRs? The nonsynonymous fixed differences in *LWS* cause 7-nm shifts in the absorption spectra of LWS pigments [18]. The fixed differences in four genes (melanopsin A, P450 aromatase, UDP-*N*-acetylglucosamine transporter, and *aloxe3*) were located in the upstream regions of the genes and are associated with significant differences in expression between the two species. P450 aromatase expression in the anterior part of lateral skin might partly explain the difference in regulation of color pattern formation between the two species (Fig. 1a); this gene plays a regulatory role in various sexual traits [62]. The different expression levels of these four genes suggest that the fixed differences affect gene expression levels (Fig. 4). Hence, the fixed differences observed in DRs are expected to affect protein functions or gene expression. These results support the hypothesis that the divergence of Lake Victoria cichlid species is explained by differentiation in short genomic regions containing genes responsible for adaptation. Each of the short genomic regions may be responsible for the adaptive traits, and the combination of these traits including LWS adaptation may lead to reproductive isolation between ecologically different species.

It has been argued that standing genetic variation is important for the recent radiation of cichlid lineages based on genome-wide shared polymorphisms among different cichlid lineages [13, 14, 69], many of which are likely selectively neutral. Recently, Meier et al. reported that the ancient admixture event between distantly related lineages (Congolese and Upper Nile) would increase genetic variation that would have contributed to the morphological diversity and adaptive traits in the Lake Victoria region superflock [20], suggesting the importance of standing genetic variation. However, the origins of adaptive variants are not fully elucidated. We examined whether or not the origin of adaptive variants in our DRs are the same age as the two divergent lineages in Meier et al. 2017 by tracing back to the origins of the putative adaptive variants (i.e., fixed differences in DRs) in cichlid species. The phylogenetic trees based on the five DR sequences were in agreement with this prediction. Furthermore, the origins of the other 11 DRs were older than the common ancestor of the Lake Victoria region superflock. These estimations suggest that the adaptive variants in DRs can be traced back to some point in the ancestral lineage of modern haplochromines [57].

In this study, we demonstrate extensive gene flow between ecologically differentiated species, 21 DRs, and an ancient origin of the adaptive variants responsible for species divergence. These findings provide new insight into a long-standing question: why do cichlids represent the most successful radiation in Lake Victoria during a very short evolutionary period? The ancient origin of adaptive variants within DRs provides an important clue. These mutations tended to accumulate after the split of the modern haplochromine lineage, supporting the idea that the radiation of Lake Victoria species occurred via selection on standing genetic variation [20]. It may also be explained by extensive gene flow, which enables species to share functional sequences that may have promoted adaptation to various environmental conditions within Lake Victoria. Indeed, the same *LWS* allele that is adaptive to local light environments has been observed in multiple species [15, 18, 19]. In this study, we focused on two species in Lake Victoria. Additional genomic sequences of multiple individuals from additional species pairs with extensive gene flow will allow us to paint a more comprehensive picture of the role of gene flow in Lake Victoria cichlid radiation.

## Methods

### Sample information

Two Lake Victoria cichlid species were used, i.e., *Haplochromis pyrrhocephalus* Witte and Witte-Maas (1987) [70] and *H.* sp. ‘macula.’ These species are widely distributed in Lake Victoria [3, 71] and inhabit Mwaburugu at the east end of Speke Gulf (Fig. 1a). All specimens were collected in sympatry by netting (1.5-m height) at a 1- to 3-m depth in Mwaburugu. All fish were collected by M.A. and S.M. in 2004–2006. The identification of all specimens was verified by M.A. and S.M.

Species identification: *Haplochromis pyrrhocephalus* is one of the most common pelagic-sublittoral species in the eastern Speke gulf. The species is recognized to be in the *Yssichromis* group because of the slender body (body depth 27.5–31.1% of standard length in the original description and 26.2–30.3% in our measurement, see material and methods in [72]). This species is distinguished from all other Haplochromines by a combination of the slender body and male nuptial coloration: 1) orange to red coloration on the head, unpaired fins, and egg dummies, and 2) absence of a lateral band. We collected 14 species from Mwaburugu (Fig. 1a), southeastern Speke gulf, where no slender-bodied species were found except this species. Therefore, we identified slender-bodied females within the range described above as *H. pyrrhocephalus.*

*Haplochromis* sp. 'macula' was described by the *Haplochromis* Ecology Survey Team (HEST) in Leiden University and subsequently re-described by Seehausen [3] with male nuptial coloration. Male of the species is relatively easy to identify because of the bright red coloration on the head, anterior body, and dorsal fin membrane, and yellow to green coloration on the posterior body and caudal peduncle. Among all species that we collected in Mwaburugu, *H*. sp. 'macula' is morphologically different from the other species by the combination of the following traits: 1) dorsal head profile is straight or weakly moderately curved (vs. moderately curved in the other species); 2) oral teeth in outer jaw are weakly compressed (vs. cylindrical to weakly compressed); 3) flange of main cusp of oral teeth in outer jaw are relatively prominent (vs. without or weak flange); and 4) arrangement of anterior teeth in outer jaw is relatively dense, with posterior end of the tooth slightly overlapping to the anterior end of the neighboring tooth (vs. not overlapping). In this study, we chose females which possessed all of these characteristics as *H*. sp. 'macula'.

Additional genetic information: all specimens used in the present study were subjected to the species identification procedure described above prior to the analysis of opsin genes [18]. Among all species that we collected in Mwaburugu, one *LWS* gene allele Py and H was exclusively fixed in *H. pyrrhocephalus* and *H*. sp. 'macula', respectively. In particular, the Py allele was only found in *H. pyrrhocephalus*. Thus, these two species possess a genetic biomarker specific to species. Note, however, that we did not identify these species by using genetic information.

### Pooled genomic DNA sequencing (Pool-seq) and mapping

Genomic DNAs were extracted from caudal or pectoral fins of wild-caught individuals using the DNeasy Blood & Tissue Kit (Qiagen, Hilden, Germany). All tissues were dissected and kept in 100% ethanol until use. Equal amounts of DNAs (500 ng) extracted from 10 males and 10 females each from *H. pyrrhocephalus* and *H*. sp. ‘macula’ were pooled. In each species, DNAs from males and females were pooled separately. Libraries were constructed using the TruSeq DNA LT Sample Prep Kit (Illumina, San Diego, CA, USA) and the sequences were determined (paired-end 100 bp) using the Illumina HiSeq2000 platform. The paired-end short reads were mapped to the reference genome sequence of *Pundamilia nyererei* [14] using Bowtie 2 [73] specifying “--score-min L,0,-0.2.”

The number of high-quality alleles was counted at every site using Samtools with the arguments “-C50 -q20 -Q30” [ver. 0.1.19-44,428 cd; [74]]. If an indel was called, the site was filtered out, including the regions 9 bp upstream and downstream of the site. Sites with ≥3 nucleotides were removed. Sites with coverage of ≥20× were retained. Finally, if PV4 information was available, a site was filtered out if the *P*-value for strand bias or tail distance bias was less than 10^− 4^ or if the *P*-value for baseQ bias was less than 10^− 100^ according to the default settings in VCFtools [75].

### Population genomic analyses

Pool-seq data for each species were initially analyzed separately. Mono- and bi-allelic sites with coverage of 80–200× were screened for each species to calculate the site frequency spectrum (SFS) by applying the EM algorithm developed by Boitard et al. [42] (Additional file 2: Text S1). The demographic history of each species was inferred from the SFS using δa/δi [46] (Additional file 2: Text S1).

Population differentiation between the two cichlid species was analyzed. Sites with coverage of 20–200× were extracted for both species, and the allele frequency was estimated at every site by applying Eq. (1) in [42]:

3 $$ \mathrm{P}\left({\mathrm{Z}}_i\;|\;{Y}_i\right)=\underset{j:{Z}_{i,j}=1}{\Pi}\;\left(\left(1-\varepsilon \right)\;\frac{Y_i}{n}+\varepsilon \left(1-\frac{Y_i}{n}\right)\;\right)\times \underset{j:{Z}_{i,j}=0}{\Pi}\;\left(\left(1-\varepsilon \right)\;\left(1-\frac{Y_i}{n}\right)+\varepsilon \frac{Y_i}{n}\;\right) $$

where *Y*_i_ is the number of derived alleles at the *i*th genomic position in *n* sampled chromosomes, *Z*_i_ is the observed reads at the *i*th position with coverage *r*_i_, *Z*_i,j_ (1 ≤ *j* ≤ *r*_i_) is an indicator variable equal to 1 if the *j*th read has the derived mutation, and 0 otherwise, and ε is the error rate of sequencing (Additional file 2: Text S1). Because we did not know the ancestral state of alleles, we used *P*_f_(*Z*_i_|*Y*_i_) = 1/2*P*(*Z*_i_|*Y*_i_) + 1/2*P*(1-*Z*_i_|*Y*_i_) to fold the SFS. We estimated the SFS as the probability is maximized. We also used the Eq. (3) to estimate an allele frequency at every site (Additional file 2: Text S1). A genome scan was performed to identify highly differentiated genomic regions. We performed a sliding window analysis in 10-kb windows with 5-kb increments after discarding windows, in which < 50% of sites were covered by ≥20× reads. We calculated the *F*_ST_ value in each window and screened windows with the top 0.1% of *F*_ST_ values. A neutrality test was performed using the ms software [76]. A coalescent simulation of 80 chromosomes was performed given the inferred population expansion (Additional file 2: Text S1). The length of the simulated region was set to 10 kb, which was the same as the window size used for the genome scan. The 80 chromosomes were randomly divided into subsamples of 40 to simulate a panmictic population. Then, Pool-seq data were simulated, the allele frequency was estimated for each site, and *F*_ST_ was calculated. These processes was repeated 100,000 times. To maximize the variance of the null distribution of *F*_ST_, we assumed no recombination and set coverage to 20× for both species.

### The effect of divergent selection under the pressure of migration

We assumed an isolation with migration model with two populations (populations 1 and 2) with population size, *N*, into which we incorporated mutation, recombination, migration, divergent selection, and random genetic drift. We consider a two-locus biallelic model. The locus I has *A* and *a* alleles and is the target of divergent selection, where the fitness values of *A* and *a* in population 1 are 1 and 1–*s*, and those in population 2 are 1–*s* and 1, and *s* is the selection coefficient. We assume that divergent selection acts in an additive manner. The locus II has *B* and *b* alleles with no phenotypic effect. Symmetric mutation occurs only in locus II at rate μ per generation to measure the hitchhiking effect of divergent selection. Recombination between the two loci is incorporated at rate *r* per generation. Symmetric migration occurs at the rate *m* per gamete per generation between the populations. Let the frequencies of *A*-*B*, *A*-*b*, *a*-*B*, and *a*-*b* in population 1 be *x*_1_, *x*_2_, *x*_3_, and *x*_4_, respectively. As such, let those in population 2 be *y*_1_, *y*_2_, *y*_3_, and *y*_4_, respectively. The expectations of these frequencies in the next generation can be given by the following recursion equations:

3a $$ {x_1}^{'}=\left(1-\upmu \right)\ {x}_1+\upmu\ {x}_2-r\ {D}_{\mathrm{x}}-m\ \left({x}_1-{y}_1\right)+s\ {x}_1\ \left({x}_3+{x}_4\right) $$

3b $$ {x_2}^{'}=\left(1-\upmu \right)\ {x}_2+\upmu\ {x}_1+r\ {D}_{\mathrm{x}}-m\ \left({x}_2-{y}_2\right)+s\ {x}_2\ \left({x}_3+{x}_4\right) $$

3c $$ {x_3}^{'}=\left(1-\upmu \right)\ {x}_3+\upmu\ {x}_4+r\ {D}_{\mathrm{x}}-m\ \left({x}_3-{y}_3\right)-s\ {x}_3\ \left({x}_1+{x}_2\right) $$

3d $$ {x_4}^{'}=\left(1-\upmu \right)\ {x}_4+\upmu\ {x}_3-r\ {D}_{\mathrm{x}}-m\ \left({x}_4-{y}_4\right)-s\ {x}_4\ \left({x}_1+{x}_2\right) $$

3e $$ {y_1}^{'}=\left(1-\upmu \right)\ {y}_1+\upmu\ {y}_2-r\ {D}_{\mathrm{y}}-m\ \left({y}_1-{x}_1\right)-s\ {y}_1\ \left({y}_3+{y}_4\right) $$

3f $$ {y_2}^{'}=\left(1-\upmu \right)\ {y}_2+\upmu\ {y}_1+r\ {D}_{\mathrm{y}}-m\ \left({y}_2-{x}_2\right)-s\ {y}_2\ \left({y}_3+{y}_4\right) $$

3g $$ {y_3}^{'}=\left(1-\upmu \right)\ {y}_3+\upmu\ {y}_4+r\ {D}_{\mathrm{y}}-m\ \left({y}_3-{x}_3\right)+s\ {y}_3\ \left({y}_1+{y}_2\right) $$

3i $$ {y_4}^{'}=\left(1-\upmu \right)\ {y}_4+\upmu\ {y}_3-r\ {D}_{\mathrm{y}}-m\ \left({y}_4-{x}_4\right)+s\ {y}_4\ \left({y}_1+{y}_2\right) $$

where *D*_x_ = *x*_1_ *x*_4_ – *x*_2_ *x*_3_ and *D*_y_ = *y*_1_ *y*_4_ – *y*_2_ *y*_3_.

We simulated the pattern of polymorphisms around the target site of divergent selection. We fixed *N* = 1000, and 4*N*μ = 0.01. We used a wide range of 4*Nr* to be 0.1~200. We assumed that the locus I is monomorphic for *A* in both populations, and performed a pre-run until the DNA polymorphism in locus II reached a mutation-drift equilibrium using the Eq. (3a, b, c, d, e, f, g, h, i). At time *T* = 0, we introduced *a* in population 2 with the initial frequency, 1/2 *N*, and ran the simulation for 8 *N* generations. We ran simulations for 100,000 cycles each with the variety of pairs of *m* and *s*, and calculated expected π_W_, π_B_, and *F*_ST_ in locus II at several time points.

### RNA-seq and assembly

To screen the candidates of differentially expressed genes between the two species, a pooled RNA-seq analysis was performed. Total RNAs were extracted from the anterior part of the lateral skins of five males each from *H. pyrrhocephalus* and *H*. sp. ‘macula.’ Equal amounts of total RNAs (1 μg) were pooled, libraries were constructed using the TruSeq RNA Library Preparation Kit (Illumina), and the sequences were determined (paired-end 100 bp) using the Illumina HiSeq2000 platform. De novo assembly of paired-end short reads (7.7 Gbp) of *H*. sp. ‘macula’ was performed using the CLC genomic workbench (https://www.qiagenbioinformatics.com/) with automatic word size. The short reads from both species (*H. pyrrhocephalus*, 6.3 Gbp; *H*. sp. ‘macula,’ 7.7 Gbp) were mapped to the assembled sequences (50,240 contigs) and the expression levels of sequences were compared between species using the CLC genomic workbench. The sequences with different expression between species (*t*-test with Bonferroni correction, *P* < 0.05) were differentially expressed candidate genes. In total, 50,240 contigs were tested and 172 (0.3%) showed differential expression. For gene identification, the differentially expressed contig sequences were subjected to a BLASTN search against the NCBI non-redundant nucleotide sequences database (https://www.ncbi.nlm.nih.gov/). The differentially expressed contigs that were found in DRs were selected as candidate genes for differential expression between the two species.

### Real-time qPCR

The expression of the candidate genes for differential expression screened by pooled RNA-seq were further analyzed by real-time qPCR (qPCR) between laboratory-reared individuals of *H*. sp. ‘macula’ and *H. pyrrhocephalus*. Fishes were 9–12 months old and were kept at 25 °C under commercial fluorescent lights with a 12 h light-dark cycle. To sample eye tissues, six individuals each from both species were euthanized under anesthesia using ethyl 4-aminobenzoate at 10 h after the light was turned on, and right eyes were enucleated. The eyes were immediately placed on ice in RNAlater (Ambion, Austin, TX, USA) and the cornea and lens were removed. The remaining eye samples were stored in fresh RNAlater at ˗80 °C until further use. To sample skin tissues, 10 individuals (five males and five females) each from both species were euthanized as described above at 5 h after the light was turned on. The euthanized fishes were immediately placed on ice in RNAlater and subsequent dissection was performed in this solution. A square area of the anterior part of the lateral skin was dissected. After muscle attached to the dissected skin was removed, the skin was cut into 2–5 mm^2^ pieces. The skin pieces were stored in fresh RNAlater at ˗80 °C until further use.

Total RNA was extracted from the eye and skin samples using TRIzol RNA Isolation Reagent (Thermo Fisher Scientific, Waltham, MA, USA) according to the manufacturer’s instructions and quantified using a NanoDrop 2000c spectrophotometer (Thermo Fisher Scientific). First-strand cDNA was reverse-transcribed from 500 ng of the eye total RNA or 1 μg of the skin total RNA using a PrimeScript RT Reagent Kit with gDNA Eraser (TaKaRa). The eye cDNA samples were diluted 25-fold in PCR-grade water for the amplification of melanopsin A. The skin cDNA samples were diluted 1.5- or 20-fold in PCR-grade water for the amplification of *aloxe3* and UDP-*N*-acetylglucosamine transporter or for *P450*, respectively. Target genes and an internal control gene (*GAPDH*) were amplified from the cDNA samples in a 25-μl total volume of PCR solution containing 12.5 ml of SYBR *Premix Ex Taq* II (TaKaRa), 3 ml of the diluted cDNA samples, and 10 pmol each of the following forward and reverse primers: melanopsin A: 5′ − TGGAGCTTTCATCGATGGCTACAAC− 3′ and 5′ − GATGCCTACAGCAAGGATGACAACAC− 3′; *GAPDH*: 5′ − GCCCACGCAAACATCATTC− 3′ and 5′ − GTCAGATCCACCACTGACACATC− 3′; *aloxe3*: 5′ − GAAGCTGCAAGGTGACAGGACTATTG− 3′ and 5′ − TGAGATGGTCAAGTTCGTCACCATG− 3′; *P450*: 5′ − GAGAAATCTGAACGCAGACTGCAAAC− 3′ and 5′ − GGACAGCAGTGACTTCTGATGCTCTATC− 3′; UDP-*N-*acetylglucosamine transporter: 5′ − AGCGAGGACAGGACCATCAAGAG− 3′ and 5′ − GAGACACGTATTTTAGCCTGGAGGAAAG− 3′. PCRs were performed using the Thermal Cycler Dice Real Time System II (TaKaRa) with the following conditions: 95 °C for 30 s, followed by 40 cycles of 95 °C for 5 s and 60 °C for 30 s. Correction of the PCR efficiency for each primer set was performed using a standard curve drawn from the dilution series of the cDNA samples. *GAPDH* was used as an internal control. Each sample was measured at least two times for technical replicates.

### DR sequence determination and phylogenetic tree construction

To confirm the fixed differences in differentiated regions (DRs), sets of primers were designed for four DRs (DR11, DR12, DR17, and DR19) to amplify regions including fixed differences. The primer sequences are listed below. The primers for DR5 were reported previously [15, 16]. Five DRs were amplified by PCR with the following conditions: a denaturation step for 3 min at 94 °C followed by 30 cycles of denaturation for 1 min at 94 °C, annealing for 30 s at 55 °C, and extension for 30 s at 72 °C. PCR products were purified and the sequences were determined using the Applied Biosystems Automated 3130xl Sequencer (Applied Biosystems, Waltham, MA, USA).

To construct phylogenetic trees, the sequences of DRs (~ 1 kb) and orthologous sequences from genome sequence data were used. Sets of primers were designed for 16 DRs (the remaining DRs failed to amplify), and these were amplified by PCR with the following reaction conditions: a denaturation step for 3 min at 94 °C followed by 30 cycles of denaturation for 1 min at 94 °C, annealing for 30 s at 55 °C, and extension for 1.5 min at 72 °C. The primer sequences are listed below. PCR products were cloned into the T-Vector pMD20 vector (Takara, Shiga, Japan) and the sequences were determined using the Applied Biosystems Automated 3130xl Sequencer. The genomic DNAs used as templates for amplification were the Lake Victoria species *H. pyrrhocephalus*, *H*. sp. ‘macula,’ and *H. piceatus*; riverine species *H.* sp. ‘katonga’, *H.* sp. ‘kitilda-rukwa’, and *H.* sp. ‘muzu’ (see Additional file 4: Figure S4F for localities); Lake Malawi species *Labidochromis caeruleus*, *Melanochromis auratus*, *Labeotropheus trewavasae*, *Pseudotropheus lombardoi*, and *Dimidiochromis strigatus*; and Lake Tanganyika species *Tropheus moorii*, *T. duboisi*, *T. brichardi*, *Simochromis pleurospilus*, *Petrochromis macrognathus*, *Cyprichromis coloratus*, *Ectodus descampsi*, *Perissodus eccentricus*, and *Neolamprologus tretocephalus*. The consensus sequences of the genomes of *H. pyrrhocephalus* and *H*. sp. ‘macula’ were constructed from the mapping results of the paired-end short reads to the reference genome sequence of *P. nyererei* [14, 76] using the CLC genomic workbench. Orthologous sequences of DR sequences were obtained by BLASTN searches [77] and from genome sequence data for *Pundamilia nyererei*, *Metriaclima zebra*, *Astatotilapia burtoni*, *Neolamprologus brichardi*, and *Oreochromis niloticus* [14], and consensus sequences of *H. pyrrhocephalus* and *H*. sp. ‘macula.’ In the case of DR5, upstream (2 kbp) and downstream (2 kbp) sequences of *LWS* were determined following methods described in previous studies [15, 18] and using sequences from previous studies [15, 17, 18] deposited in databases. Each of the DR sequences was aligned and subjected to a phylogenetic analysis using the maximum likelihood method with 1000 bootstrap replications in MEGA ver. 6 [78].

### Gene ontology analysis

The sequences of DRs (14–28 kbp) were used as queries for BLASTN searches [77] against the NCBI nucleotide database (http://blast.ncbi.nlm.nih.gov). The sequences of genes in DRs were subjected to a gene ontology analysis using DAVID [79] and Blast2GO [80]. The details of the Gene Ontology Analysis and primer sequences can be found in Supplemental Material online.

## Additional files

Additional file 1: **Figure S1.** (A) Site frequency spectrum of Lake Victoria cichlids. The white and black bars represent *H. pyrrhocephalus* and *H*. sp. ‘macula,’ respectively. (B) Demographic model of Lake Victoria cichlids. See the section Demographic Model and Parameter Estimation for details. (C) Nucleotide and indel frequencies within five DRs. We amplified and determined the sequences, including fixed differences, of four DRs from 20 individuals each of *H.* sp. ‘macula’ and *H. pyrrhocephalus*. Positions indicate the positions from the first nucleotides of the determined sequences. The frequencies of nucleotides in the coding region of *LWS* were verified in a previous study (*14*). (PDF 155 kb)

Additional file 2 **Text S1.** Supporting Text. (DOCX 572 kb)

Additional file 3 **Figure S2.** Population genomic analyses using only male individuals. (A) Site frequency spectrum in *H. pyrrhocephalus* (white bars; 126,843 SNPs) and *H*. sp. ‘macula’ (black bars; 146,456 SNPs). Nucleotide diversity and Tajima’s *D* values were almost same as those in all samples. (B) Average *F*ST values (±1 SD) against coverage for Pool-seq data as in Fig. 1b (5,662,990 SNPs). The blue and orange dots represent observed and simulated values under panmixia. (C) The spatial patterns of average nucleotide diversity within each species (πW; pink), average pairwise nucleotide divergence between species (πB; blue), and *F*ST (green) in and around *LWS* gene as in Fig. 2b. The green arrows represent fixed nucleotide differences between species. (PDF 279 kb)

Additional file 4: **Figure S3.** The spatial patterns of average nucleotide diversity within species (πW; pink), average pairwise nucleotide divergence between species (πB; blue), and *F*ST (green) in and around DRs. The green arrows represent fixed nucleotide differences between species. (PDF 1356 kb)

Additional file 5: **Figure S4.** The origins of mutations in DRs. Three phylogenetic trees represent the accumulation of mutations in the common ancestral species of (A) Lake Victoria species, (B) Lake Victoria and riverine *Haplochromis* species, and (C) tribe Tropheini in Lake Tanganyika, Lakes Malawi, Victoria, and riverine *Haplochromis* species. The tree topologies constructed from sequences of each DR were consistent with (D) “Riverine origin” or (E) “Modern haplochromine origin.” Scale bars indicate the number of substitutions per site. (F) The LWS sequences were determined from three riverine species: *H.* sp. ‘katonga’ from Katonga, *H.* sp. ‘kitilda-rukwa’ from Kitilda-Rukwa, and *H.* sp. ‘muzu’ from Muzu. (PDF 554 kb)

Additional file 6: **Table S1.** Genes in DRs. (PDF 149 kb)

## Abbreviation

SFS: site frequency spectrum
