# Supplementary material for: Patterns of genomic differentiation between two Lake Victoria cichlid species, Haplochromis pyrrhocephalus and H. sp. ‘macula’
Source: BMC Evol Biol. 2019 Mar 4;19:68. doi: 10.1186/s12862-019-1387-2 (PMC6399900; doi:10.1186/s12862-019-1387-2)
Supplement: Supplementary file 1 — Figure S1. (A) Site frequency spectrum of Lake Victoria cichlids. The white and black bars represent H. pyrrhocephalus and H. sp. ‘macula,’ respectively. (B) Demographic model of Lake Victoria cichlids. See the section Demographic Model and Parameter Estimation for details. (C) Nucleotide and indel frequencies within five DRs. We amplified and determined the sequences, including fixed differences, of four DRs from 20 individuals each of H. sp. ‘macula’ and H. pyrrhocephalus. Positions indicate the positions from the first nucleotides of the determined sequences. The frequencies of nucleotides in the coding region of LWS were verified in a previous study (14). (PDF 155 kb) [file 12862_2019_1387_MOESM1_ESM.pdf]

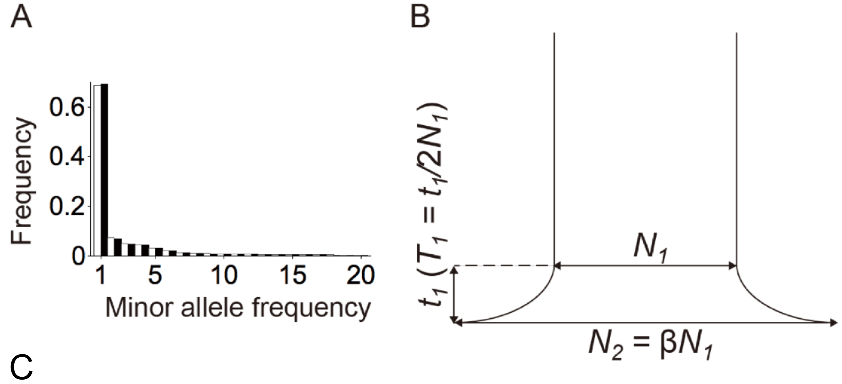

C

DR11

Positions	20	22	150
H. sp. 'macula'	T: 100%, C: 0%	T: 100%, C: 0%	A: 100%, G: 0%
H. pyrrhocephalus	T: 5%, C: 95%	T: 5%, C: 95%	A: 0%, G: 100%

DR12

Positions	27	47	54
H. sp. 'macula'	T: 95%, C: 5%	A: 5%, G: 95%	A: 95%, T: 5%
H. pyrrhocephalus	T: 0%, C: 100%	A: 100%, G: 0%	A: 0%, T: 100%

DR17

Position	92
H. sp. 'macula'	A: 100%, G: 0%
H. pyrrhocephalus	A: 0%, G: 100%

DR19

Positions	19-21	112-141	142	219	222
H. sp. 'macula'	3 bp ins.: 100%	29 bp del.: 100%	C: 100%, T: 0%	C: 100%, T: 0%	C: 100%, T: 0%
H. pyrrhocephalus	3 bp del.: 100%	29 bp ins.: 100%	C: 0%, T: 100%	C: 0%, T: 100%	C: 0%, T: 100%

DR5 (LWS)

Positions (nonsynonymous)	410	647	688	814	823, 824
H. sp. 'macula'	C: 100%, G: 0%	A: 100%, T: 0%	G: 100%, A: 0%	G: 100%, A: 0%	TG: 100%, AT: 0%
H. pyrrhocephalus	C: 0%, G: 100%	A: 0%, T: 100%	G: 0%, A: 100%	G: 0%, A: 100%	TG: 0%, AT: 100%

Figure S1. (**A**) Site frequency spectrum of Lake Victoria cichlids. The white and black bars represent *H. pyrrhocephalus* and *H.* sp. 'macula,' respectively. (**B**) Demographic model of Lake Victoria cichlids. See the section Demographic Model and Parameter Estimation for details. (**C**) Nucleotide and indel frequencies within five DRs. We amplified and determined the sequences, including fixed differences, of four DRs from 20 individuals each of *H.* sp. 'macula' and *H. pyrrhocephalus*. Positions indicate the positions from the first nucleotides of the determined sequences. The frequencies of nucleotides in the coding region of *LWS* were verified in a previous study (*14*).
