# Supplementary material for: Patterns of genomic differentiation between two Lake Victoria cichlid species, Haplochromis pyrrhocephalus and H. sp. ‘macula’
Source: BMC Evol Biol. 2019 Mar 4;19:68. doi: 10.1186/s12862-019-1387-2 (PMC6399900; doi:10.1186/s12862-019-1387-2)
Supplement: Supplementary file 2 — Text S1. Supporting Text. (DOCX 572 kb) [file 12862_2019_1387_MOESM2_ESM.docx]

Supporting Text

**Estimation of the Rate of Sequence Errors**

The rate of sequence errors was estimated based on an idea proposed by Kofler et al. [1]. The idea is that a very small read count for a minor allele is likely to be a sequence error when coverage is sufficiently high. Let *r*_minor_ and *r*_major_ be the numbers of minor and major allele reads, such that *r* = *r*_minor_ + *r*_major_ reads at a site. Consider the situation in which *r* = 200, *r*_minor_ = 1, and *r*_major_ = 199, and the number of sampled chromosomes, *n*, is 40. When the true allele frequency at a site is 1/40 or 2/40, the probability of *r*_minor_ = 1, *P*(*r*_minor_ = 1|*r*) is 0.0324 or 0.000369, respectively. However, when the site is monomorphic and the error rate, ε, is 0.001, *P*(*r*_minor_ = 1|*r*, ε) = 0.164. Thus, even when the error rate is very low, *r*_minor_ = 1 is unlikely without errors.

Utilizing this feature, ε was estimated using sites with *r* = 200–400 and *r*_minor_ = 0 or 1. The true minor allele frequencies at the sites were assumed to be 0/40 or 1/40 because *P*(*r*_minor_ = 0 or 1|*r*, ε) is very low when the frequency is ≥2/40 at sites with high coverage. Note that, if the true minor allele frequencies were 0/40 to 2/40, almost the same results are obtained.

It was necessary to estimate the proportion of sites with a minor allele frequency of 0/40 (denoted *m*) as well as ε. Thus, the joint likelihood function of ε and *m* was developed. When the frequency of a minor allele is 0/40, the proportions of major and minor alleles are (1–ε) and ε, respectively. For a frequency of 1/40, these proportions are and , respectively. Thus, *P*(*r*_minor_|*r*, ε, *m*) was obtained as follows:

(1)

Let *n*(*r*, *r*_minor_) be the observed number of sites with a given *r* and *r*_minor_. Using equation (1), the log-likelihood of ε and *m* given observed *n*(*r*, *r*_minor_) is as follows:

(2)

where *L_r_* = *n*(*r*, 0) + *n*(*r*, 1).

Using equation (2), ε was estimated as 0.00152 and 0.00155 for *H. pyrrhocephalus* and *H*. sp. ‘macula,’ respectively. In the mapping procedure, the minimum base quality score was set at 30, which was roughly equal to the error rate, 0.001. Thus, our estimate was feasible.

**Calculating Population Genetic Statistics**

Mono- and bi-allelic sites with coverages of 80–200× were screened for each species to calculate population genetic statistics. The site frequency spectrum (SFS) was inferred for each species by applying the EM algorithm developed by Boitard et al. [2] using the rate of sequence errors, ε, estimated in the previous section. This method assumes an infinite-sites model.

*Y*_i_ denoted the number of derived alleles at the *i*th genomic position (0 ≤ *Y*_i_ ≤ *n*), where *n* is the number of sampled chromosomes, and (1 ≤ *i* ≤ *M*), where *M* is the number of sites. Let *Z*_i,j_ (0 ≤ *j* ≤ *r*_i_, where *r*_i_ is sequence coverage) be an indicator variable equal to 1 if the *j*th read has the derived mutation, and 0 otherwise. The conditional probability of the observed read *Z*_i_ given *Y*_i_ is as follows:

(3)

Let *p* = (*p*_0_,..., *p*_n_) be the SFS and . The likelihood given *p* was calculated as follows:

(4)

Denoting *p*^c^ as the SFS in the *c*th step of the EM algorithm, *p*^c+1^ was calculated as follows:

(5)

Lacking an appropriate outgroup, the spectrum was folded by *P*_f_(*Z*_i_|*Y*_i_) = 1/2*P*(*Z*_i_|*Y*_i_) + 1/2*P*(1-*Z*_i_|*Y*_i_) instead.

The EM algorithm was initiated as *p*^0^, which is a flat distribution. Then, *p* and a log-likelihood value were calculated in the next iteration. The EM step continued until log *L*(*p*^c+1^) - log *L*(*p*^c^) < *M* × 10^-5^. The number of segregating sites (*S*), nucleotide diversity (θ_π_; [3]), Watterson’s θ_W_ [4], and Tajima’s *D* [5] were calculated from the inferred SFS, *p*.

The minor allele frequency was also estimated at every site. If the proportion of reads with a minor allele *p*_m_ was <0.1, the likelihood was calculated given *Y*_i_ using equation (3) and the frequency with the maximum likelihood was used. This is because the frequency of rare alleles is critically affected by sequence errors. If *p*_m_ ≥ 0.1, the closest integer to *p*_m_ × *n* was the minor allele frequency.

**Demographic Model and Parameter Estimation**

A simple population expansion model was assumed, as illustrated in Fig. S1B. The ancestral diploid population representing a river population was assumed to follow a standard Wright–Fisher model with a constant size, *N*_1_. The population expansion started *t*_1_ generations ago. This event represents the immigration of cichlid fishes into Lake Victoria from the river population [6]. The population size grew exponentially, and finally reached *N*_2_ at present. The time and population size were scaled by the ancestral population size and *T*_1_ = *t*_1_/2*N*_1_ and β = *N*_2_/*N*_1_ were estimated. The expected SFS given *T*_1_ and β was estimated, and the log-likelihood based on the observed and expected SFS was obtained using δaδi software [7]. The estimated values of *T*_1_ and β were 0.69 and 188 for *H. pyrrhocephalus*, and 0.67 and 227 for *H*. sp. ‘macula,’ respectively.

Next, the parameters *N*_1_, *N*_2_, and *t*_1_ were estimated assuming a mutation rate per site per generation of μ = 6.6 × 10^-8^ [8]. The expected coalescent time was calculated under our model following Slatkin and Hudson [9].

The population size at time *t* (*t* < *t*_1_) was *N*(*t*) = *N*_2_ e^–^*^rt^*, where the growth rate of the population per generation was *r* = –ln(1/β)/*t*_1_. First, the probabilities of the coalescent times *t* < *t*_1_ and *t* ≥ *t*_1_ were estimated. Following the model, the following were estimated:

(6)

(7)

Next, a conditional expected coalescent time was calculated. When *t* < *t*_1_, using equation (5) in Slatkin and Hudson [9], the conditional probability distribution of *t* was calculated as follows:

(8)

Then, the following was obtained:

(9)

where α = 1/2*rN*_2_, γ = exp(*rt*_1_), and *Ei*(.) is the exponential integral.

In the Additional6_textS1 same way, the probability distribution and expected coalescent time given *t* ≥ *t*_1_ were obtained as follows:

(10)

Finally, the expected coalescent time, *t*_C_, was obtained from equations (6)–(10),

(11)

Expected nucleotide diversity was calculated as 2*t*_C_μ. The parameters *N*_2_, *t*_1_, and *r* were functions of *N*_1_. Thus, 2*t*_C_μ was a function of *N*_1_. Using the observed values of nucleotide diversity, as well as and were calculated for *Haplochromis*. , , and were 17,505, 3.3 × 10^6^, and 24,157 for *H. pyrrhocephalus*, and 16,669, 3.8 × 10^6^, and 22,337 for *H*. sp. ‘macula’, respectively.

**Simulating Pool-seq Data under Panmixia**

A joint SFS was calculated under panmixia from *p* inferred as described in “Calculating population genetic statistics.” Let *c*_1i_, *c*_2i_, and *n*_cmin, cmax_ be the Pool-seq coverage at the *i*th site in species 1, that in species 2, and the number of SNPs in a bin of coverage from *c*_min_ to *c*_max_ (*c*_min_ ≤ *c*_1i_, *c*_2i_ < *c*_max_). Pool-seq data were simulated as follows: 1) a SNP was randomly sampled from the joint SFS. 2) Pool-seq data were simulated such that one allele was randomly selected given the allele frequencies, *c*_1i_ times for species 1 and *c*_2i_ times for species 2. In this process, sequence errors were incorporated at the rate ε, which was estimated as described in “Estimation of the rate of sequence errors.” 3) Allele frequencies were estimated as described in “Calculating population genetic statistics” and *F*_ST_ was calculated. 4) Steps 1) to 3) were repeated for all *n*_cmin, cmax_ SNPs.

**Repeat Identification**

To annotate repetitive regions in the *P. pundamilia* genome, RepeatModeler (ver. 1.0.8) was used to generate a species-specific repeat library. In this process, RMBlast was used as a sequence search engine. Then, RepeatMasker (ver. 4.0.5) was run using the consensus repeat libraries. The results were filtered out if a hit region in the genome covered <70% of the total length of repeats or if nucleotide divergence was >20% from the reference genome in the repeat libraries. Population genomic analyses were repeated after excluding SNPs within the repetitive regions, and the results were similar using the original and reduced datasets.

**The power to detect population differentiation by Pool-seq**

To assess the power of population differentiation by Pool-seq, we simulated coalescent processes and subsequent Pool-seq analysis with sample size, 40 in each population. We set the expected *F*_ST_ values to be 0, 0.01, and 0.05, and found that Pool-seq analysis can detect population differentiation (see Figure below).


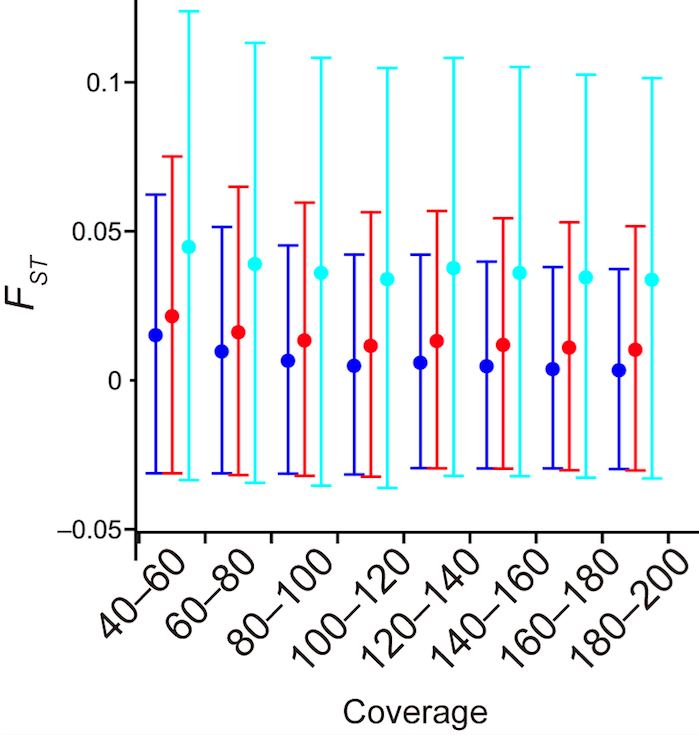


**Figure:** Average *F*_ST_ values (±1 SD) against coverage for Pool-seq data. The blue, red, and cyan dots represent simulated values with *F*_ST_, 0, 0.01, and 0.05, respectively.

**Gene Ontology Analysis**

The sequences of DRs (14–28 kbp) were used as queries for BLASTN searches [11] against the NCBI nucleotide database (http://blast.ncbi.nlm.nih.gov) to find genes. The sequences of genes in DRs were subjected to a gene ontology analysis using DAVID [12] and Blast2GO [13]. The gene ontology terms are listed below. Details of the biological functions were further investigated based on literature searches for individual genes.

| | DRs | Sequence ID | Gene name | Molecular function | Biological process | Cellular component | | --- | --- | --- | --- | --- | --- | | DR1 | XM_005724172.seq | Protein diaphanous homolog 3-like isoform x1 | Actin binding; Rho GTPase binding | Actin cytoskeleton organization | - | | DR2 | XM_005751059.seq | Ventral anterior homeobox 2-like | Sequence-specific DNA binding | Regulation of transcription, DNA-templated; Optic nerve development; Retina morphogenesis in camera-type eye; Closure of optic fissure; Regulation of retinal ganglion cell axon guidance; Negative regulation of intrinsic apoptotic signaling pathway by p53 class mediator | Nucleus | | DR3 | XM_005455229.seq | Prostaglandin D2 receptor 2-like | G-protein coupled receptor activity | G-protein coupled receptor signaling pathway | Integral component of membrane | |  | XM_005750017.seq | G-protein coupled receptor 4-like | G-protein coupled receptor activity | G-protein coupled receptor signaling pathway | Integral component of membrane | |  | XM_005750038.seq | UDP-glucuronosyltransferase 2b15-like | Transferase activity, transferring hexosyl groups | Metabolic process | - | | DR4 | XM_005749930.seq | Hemicentin-1-like isoform x2 | - | - | - | |  | XM_005939901.seq | Hemicentin-1-like isoform x1 | - | - | - | | DR5 | LWS_cd.seq | Long wavelength-sensitive opsin | G-protein coupled receptor activity; Photoreceptor activity | G-protein coupled receptor signaling pathway; Visual perception; Phototransduction; Protein-chromophore linkage | Integral component of membrane | | DR6 | XM_005453101.seq | Leucine-rich repeat and immunoglobulin-like domain-containing nogo receptor-interacting protein 1 | F-protein binding | P:axon regeneration |  | |  | XM_005735193.seq | Netrin receptor UNC5c-like isoform x2 | F-protein binding | Signal transduction | - | | DR7 | XM_005730166.seq | General transcription factor IIh subunit 1-like | - | Nucleotide-excision repair; Transcription, DNA-templated | Core TFIIH complex | | DR8 | XM_005730137.seq | Intestinal mucin-like | Peptidase inhibitor activity | Negative regulation of peptidase activity | Extracellular region | | DR9 | XM_005751143.seq | Hepatocyte growth factor receptor-like isoform x1 | Protein tyrosine kinase activity; Receptor activity; ATP binding | Neuron migration; Liver development; Peptidyl-tyrosine phosphorylation; Spinal cord motor neuron differentiation; Cerebellar granule cell differentiation; Exocrine pancreas development; Pectoral fin development; Protein binding | Integral component of membrane | | DR11 | XM_005727983.seq | AP-4 complex subunit epsilon-1 | - | Intracellular protein transport; Vesicle-mediated transport; Binding | Membrane coat | |  | XM_005727984.seq | Cytochrome p450 aromatase type II | Monooxygenase activity; Iron ion binding; Oxidoreductase activity, acting on paired donors, with incorporation or reduction of molecular oxygen; Heme binding | Response to xenobiotic stimulus; Response to estradiol; Oxidation-reduction process | - | |  | XM_005727986.seq | Gliomedin-like isoform x1 | Protein binding | - | - | | DR12 | XM_005749719.seq | Melanopsin-A-like isoform x1 | G-protein coupled receptor activity; Photoreceptor activity | G-protein coupled receptor signaling pathway; Visual perception; Phototransduction; Protein-chromophore linkage | Integral component of membrane | | DR14 | XM_005732149.seq | Myosin-2 heavy non muscle-like | - | - | - | |  | XM_005732150.seq | Pollen-specific leucine-rich repeat extensin-like protein 1-like | - | - | - | | DR15 | XR_312020.seq | ---NA--- | - | - | - | | DR16 | XM_005743315.seq | Low quality protein: aryl hydrocarbon receptor nuclear translocator-like protein 2-like | - | - | - | | DR17 | XM_004570512.seq | UDP-n-acetylglucosamine transporter-like | Nucleotide-sugar transmembrane transporter activity; Sugar: proton symporter activity | Carbohydrate transport; Nucleotide-sugar transport; Proton transport; Nucleotide transmembrane transport | Golgi membrane; Integral component of membrane | |  | XM_005720962.seq | U3 small nucleolar ribonucleoprotein protein imp3-like | rRNA binding | - | Intracellular | | DR18 | XM_005733013.seq | Peptidyl-prolyl cis-trans isomerase h-like | Peptidyl-prolyl cis-trans isomerase activity | Protein peptidyl-prolyl isomerization; Protein folding | - | |  | XM_005733015.seq | Transcription initiation factor TFIID subunit 10-like | DNA-templated transcription, initiation | - | Nucleus | |  | XM_005941175.seq | Probable G-protein coupled receptor 160 | - | - | - | | DR19 | XM_005742045.seq | Type II cytoskeletal 5-like isoform x2 | Structural molecule activity | - | Keratin filament | | DR20 | XM_005748525.seq | Hydroperoxide isomerase aloxe3-like | Iron ion binding; Oxidoreductase activity, acting on single donors with incorporation of molecular oxygen, incorporation of two atoms of oxygen | Oxidation-reduction process | - | |  | XM_005951547.seq | Macrophage mannose receptor 1-like | Carbohydrate binding | - | - | | DR21 | XM_005750946.seq | RAS-related protein rab-11a-like | GTP binding; GTPase activity | Small GTPase mediated signal transduction; Nucleocytoplasmic transport; Obsolete GTP catabolic process; Intracellular protein transport | Intracellular; Membrane | |  | XM_005750947.seq | RNA-binding protein mex3a-like | RNA binding; Zinc ion binding; Protein binding | - | - | |
| --- | --- | --- | --- | --- | --- | --- | --- | --- | --- | --- | --- | --- | --- | --- | --- | --- | --- | --- | --- | --- | --- | --- | --- | --- | --- | --- | --- | --- | --- | --- | --- | --- | --- | --- | --- | --- | --- | --- | --- | --- | --- | --- | --- | --- | --- | --- | --- | --- | --- | --- | --- | --- | --- | --- | --- | --- | --- | --- | --- | --- | --- | --- | --- | --- | --- | --- | --- | --- | --- | --- | --- | --- | --- | --- | --- | --- | --- | --- | --- | --- | --- | --- | --- | --- | --- | --- | --- | --- | --- | --- | --- | --- | --- | --- | --- | --- | --- | --- | --- | --- | --- | --- | --- | --- | --- | --- | --- | --- | --- | --- | --- | --- | --- | --- | --- | --- | --- | --- | --- | --- | --- | --- | --- | --- | --- | --- | --- | --- | --- | --- | --- | --- | --- | --- | --- | --- | --- | --- | --- | --- | --- | --- | --- | --- | --- | --- | --- | --- | --- | --- | --- | --- | --- | --- | --- | --- | --- | --- | --- | --- | --- | --- | --- | --- | --- | --- | --- | --- | --- | --- | --- | --- | --- | --- | --- | --- | --- | --- | --- | --- | --- | --- | --- | --- | --- | --- | --- | --- | --- | --- | --- | --- |

**Primer Sequences**

Primer sequences for the amplification of DR regions to verify fixed differences

|  | **Forward** | **Reverse** |
| --- | --- | --- |
|  | 5′ end to 3′ end | 5′ end to 3′ end |
| **DR11** | CTCTGCTGTTCTTTTAATCTTTAGG | GTGTCTTTAATACGATCATGTGAG |
| **DR12** | CTGAGGTATGACTAGCATTACTG | CACTCAGCTGCAGTTACTTGTG |
| **DR17** | CTTTGCACACGCTCATCAGTGT | TAAATGGAGGTATAGCCACTCAC |
| **DR19** | TTCTTGCAGTTAGAGATGCAATCG | AGTCTGTACATGCTCTGCAACTTG |

Primer sequences for the amplification of DR regions to construct phylogenetic trees

|  | **Forward** | **Reverse** |
| --- | --- | --- |
|  | 5′ end to 3′ end | 5′ end to 3′ end |
| **DR2** | TTCACTGAGCTCTCAGGTGAGT | GTCCATAATACACCTCTCTTG |
| **DR3** | ATCATGTGACACTGACTCTGTCAAG | CTCTCAAGGATGTTCTTGAGAATC |
| **DR6** | TCTATGAACAGGTTAGAGGTGT | TAAGTAACTTACCCAACACTGT |
| **DR7** | TTTGGAGCAAGTAAGTGTTATGAG | GACCAAAGTGTGAAATTGACAC |
| **DR8** | CTAACAAACAGACAGACAGAG | TAAGGAGCTGATTATTGCTGTTG |
| **DR10** | CCAGGTCACTTGATAGAACTG | GTCTGGGTCGTGTTATGTTCAG |
| **DR11** | TAACAGAGCTGAAGCAGTTAGAG | ATTCCTCTCCAACTGCTCTACTG |
| **DR12** | GTGATAGCTACATCTTTCCTACT | ATAGTTTCTCTCTGAGTGTAACTG |
| **DR14** | ACCAGCTGTGTAGTGTTCAG | GAGAAATATCAGGGTAGTCTTTG |
| **DR15** | GCCAAGTTTCCACCATGTTTG | CTTAAAACTGGCACAATGAATTCAG |
| **DR16** | CTGTAATATGGTATGTCATCTG | GATAAAGTGACAGACACGTATCTAG |
| **DR17** | TCTACTGTAACTCAGACTGTAG | TGCACAAATGACTGTCTGCAAAC |
| **DR18** | AGATAGCACAACAGCAGAAATGATG | ATGAATGTGCATGACTGCAATGAAG |
| **DR19** | AGGTGGAGATACATATGTGAAG | TAGGCTTTGCAGTGTTTGTCTG |
| **DR20** | CTTGAACACTTACAGAGTCAGAG | CAGTATTTACATGCTCCAGATAGC |

**References**

1. Kofler R, Orozco-terWengel P, De Maio N, Pandey RV, Nolte V, Futschik A, Kosiol C, Schlotterer C: **PoPoolation: a toolbox for population genetic analysis of next generation sequencing data from pooled individuals**. *PLoS One* 2011, **6**(1):e15925.

2. Boitard S, Schlotterer C, Nolte V, Pandey RV, Futschik A: **Detecting selective sweeps from pooled next-generation sequencing samples**. *Mol Biol Evol* 2012, **29**(9):2177-2186.

3. Tajima F: **Evolutionary relationship of DNA sequences in finite populations**. *Genetics* 1983, **105**(2):437-460.

4. Watterson G: **On the number of segregating sites in genetical models without recombination**. *Theoretical population biology* 1975, **7**(2):256-276.

5. Tajima F: **Statistical method for testing the neutral mutation hypothesis by DNA polymorphism**. *Genetics* 1989, **123**(3):585-595.

6. Nagl S, Tichy H, Mayer WE, Takezaki N, Takahata N, Klein J: **The origin and age of haplochromine fishes in Lake Victoria, East Africa**. *Proceedings of the Royal Society of London B: Biological Sciences* 2000, **267**(1447):1049-1061.

7. Gutenkunst RN, Hernandez RD, Williamson SH, Bustamante CD: **Inferring the joint demographic history of multiple populations from multidimensional SNP frequency data**. *PLoS Genet* 2009, **5**(10):e1000695.

8. Recknagel H, Elmer KR, Meyer A: **A hybrid genetic linkage map of two ecologically and morphologically divergent Midas cichlid fishes (Amphilophus spp.) obtained by massively parallel DNA sequencing (ddRADSeq)**. *G3 (Bethesda)* 2013, **3**(1):65-74.

9. Slatkin M, Hudson RR: **Pairwise comparisons of mitochondrial DNA sequences in stable and exponentially growing populations**. *Genetics* 1991, **129**(2):555-562.

10. Hein J, Schierup M, Wiuf C: **Gene genealogies, variation and evolution: a primer in coalescent theory**: Oxford University Press, USA; 2004.

11. Altschul SF, Gish W, Miller W, Myers EW, Lipman DJ: **Basic local alignment search tool**. *Journal of molecular biology* 1990, **215**(3):403-410.

12. Huang DW, Sherman BT, Lempicki RA: **Systematic and integrative analysis of large gene lists using DAVID bioinformatics resources**. *Nature protocols* 2009, **4**(1):44-57.

13. Conesa A, Götz S, García-Gómez JM, Terol J, Talón M, Robles M: **Blast2GO: a universal tool for annotation, visualization and analysis in functional genomics research**. *Bioinformatics* 2005, **21**(18):3674-3676.
