# Supplementary material for: Patterns of genomic differentiation between two Lake Victoria cichlid species, Haplochromis pyrrhocephalus and H. sp. ‘macula’
Source: BMC Evol Biol. 2019 Mar 4;19:68. doi: 10.1186/s12862-019-1387-2 (PMC6399900; doi:10.1186/s12862-019-1387-2)
Supplement: Supplementary file 3 — Figure S2. Population genomic analyses using only male individuals. (A) Site frequency spectrum in H. pyrrhocephalus (white bars; 126,843 SNPs) and H. sp. ‘macula’ (black bars; 146,456 SNPs). Nucleotide diversity and Tajima’s D values were almost same as those in all samples. (B) Average FST values (±1 SD) against coverage for Pool-seq data as in Fig. 1b (5,662,990 SNPs). The blue and orange dots represent observed and simulated values under panmixia. (C) The spatial patterns of average nucleotide diversity within each species (πW; pink), average pairwise nucleotide divergence between species (πB; blue), and FST (green) in and around LWS gene as in Fig. 2b. The green arrows represent fixed nucleotide differences between species. (PDF 279 kb) [file 12862_2019_1387_MOESM3_ESM.pdf]

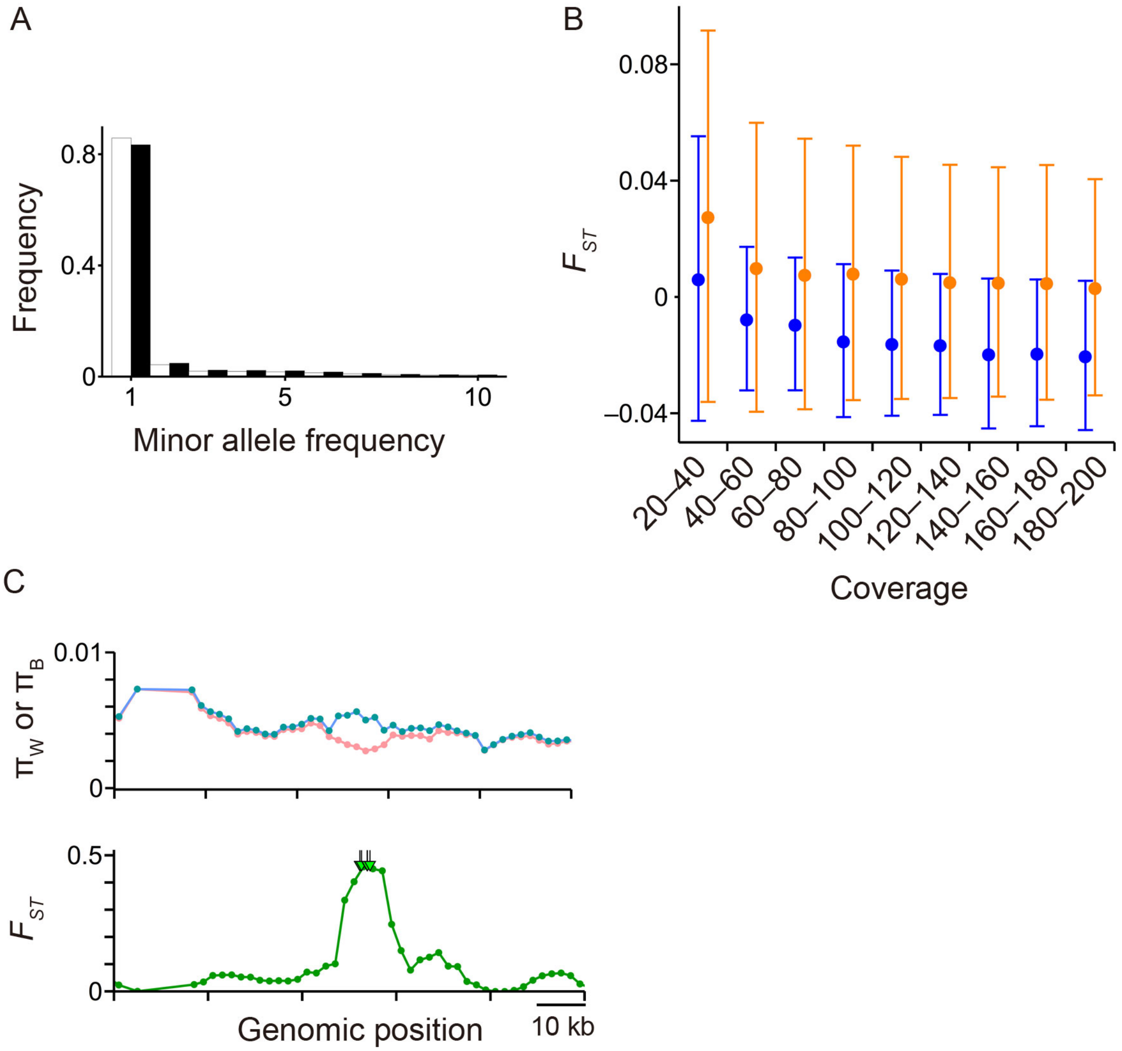

Figure S2. Population genomic analyses using only male individuals. (**A**) Site frequency spectrum in *H. pyrrhocephalus* (white bars; 126,843 SNPs) and *H.* sp. 'macula' (black bars; 146,456 SNPs). Nucleotide diversity and Tajima's *D* values were almost same as those in all samples. (**B**) Average F_{ST} values (±1 SD) against coverage for Pool-seq data as in Fig. 1B (5,662,990 SNPs). The blue and orange dots represent observed and simulated values under panmixia. (**C**) The spatial patterns of average nucleotide diversity within each species (π_W; pink), average pairwise nucleotide divergence between species (π_B; blue), and F_{ST} (green) in and around *LWS* gene as in Fig. 2B. The green arrows represent fixed nucleotide differences between species.
