# Supplementary material for: Patterns of genomic differentiation between two Lake Victoria cichlid species, Haplochromis pyrrhocephalus and H. sp. ‘macula’
Source: BMC Evol Biol. 2019 Mar 4;19:68. doi: 10.1186/s12862-019-1387-2 (PMC6399900; doi:10.1186/s12862-019-1387-2)
Supplement: Supplementary file 4 — Figure S3. The spatial patterns of average nucleotide diversity within species (πW; pink), average pairwise nucleotide divergence between species (πB; blue), and FST (green) in and around DRs. The green arrows represent fixed nucleotide differences between species. (PDF 1356 kb) [file 12862_2019_1387_MOESM4_ESM.pdf]

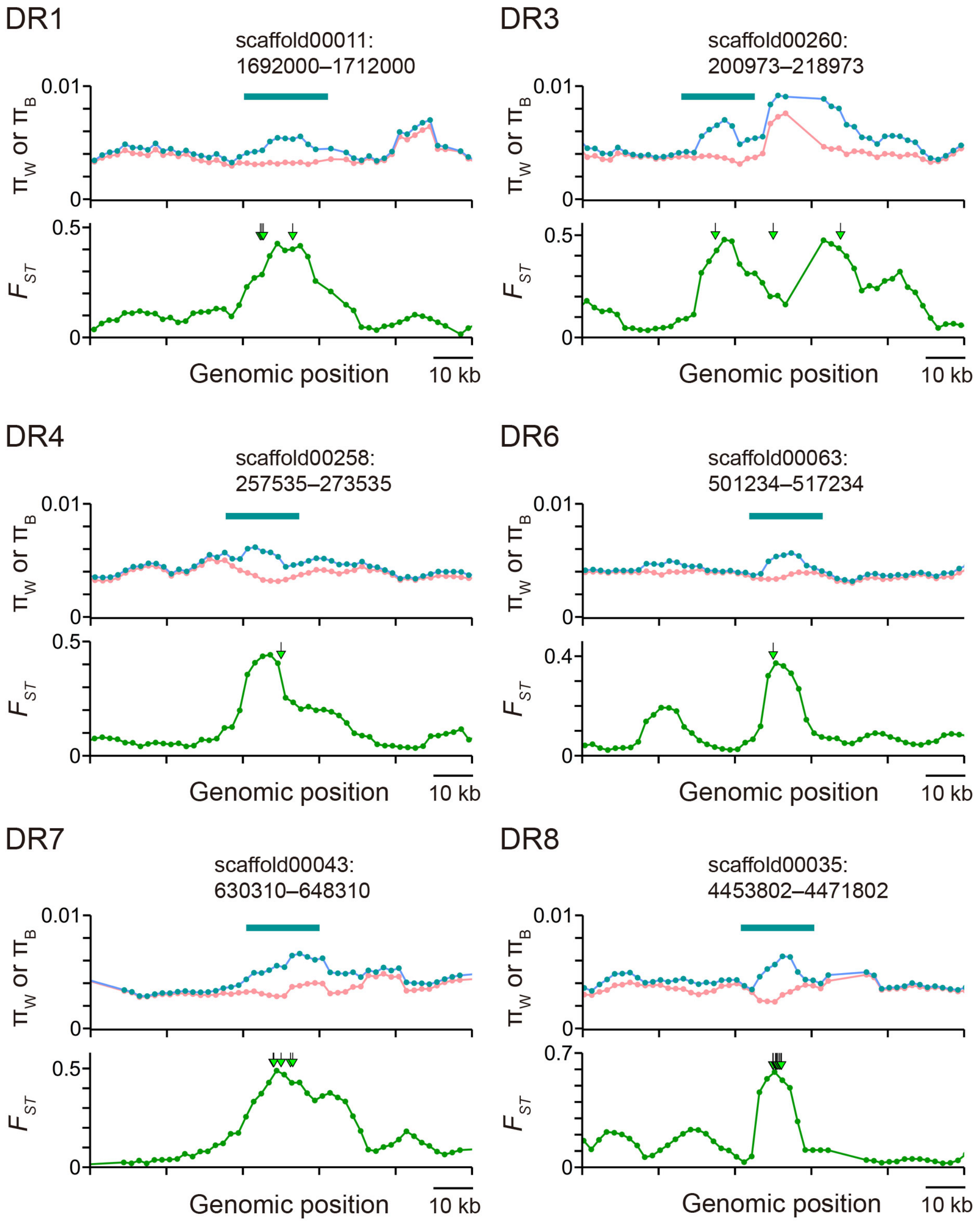

Figure S3. The spatial patterns of average nucleotide diversity within species (π_W; pink), average pairwise nucleotide divergence between species (π_B; blue), and F_{ST} (green) in and around DRs. The green arrows represent fixed nucleotide differences between species.

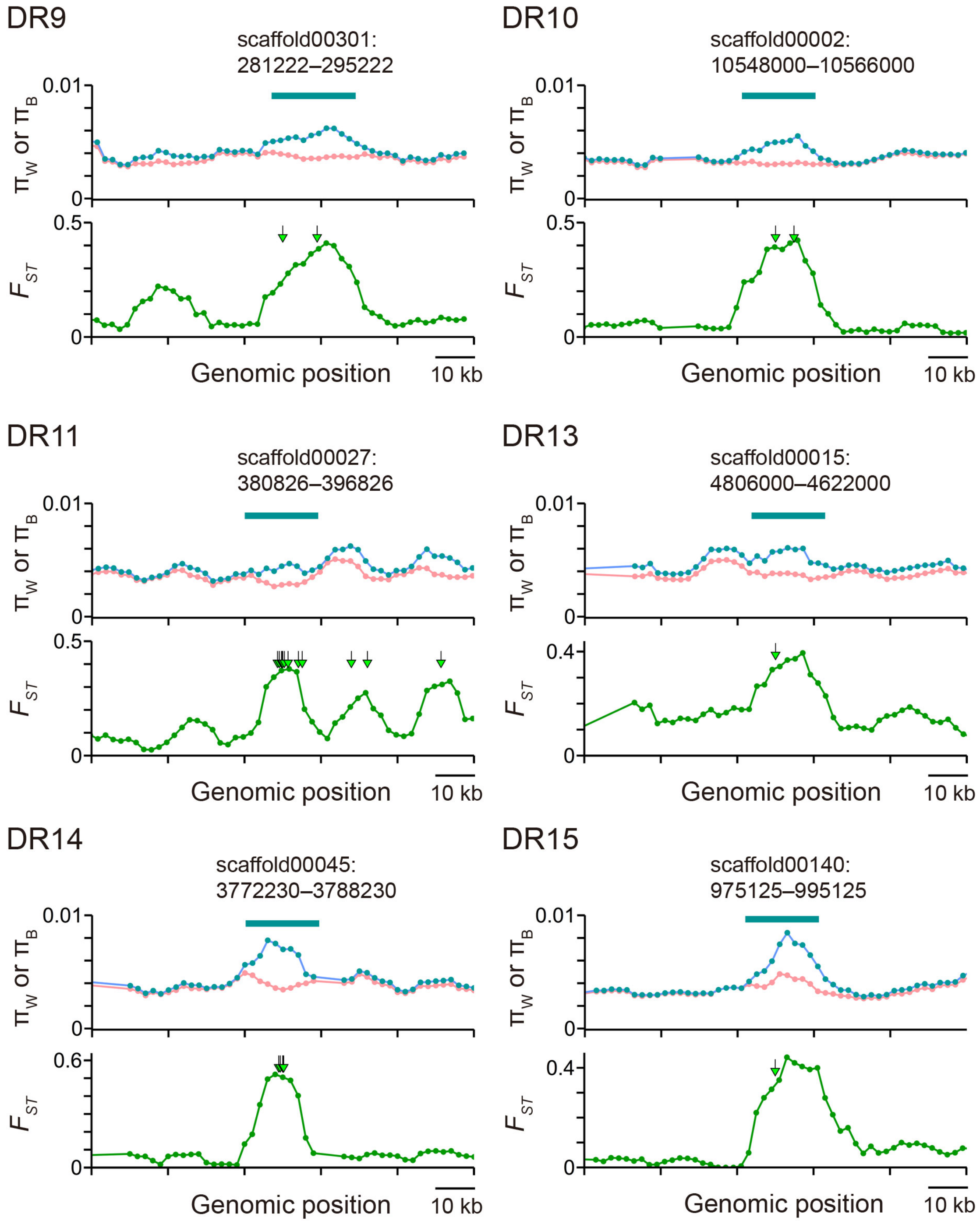

Figure S3. continued

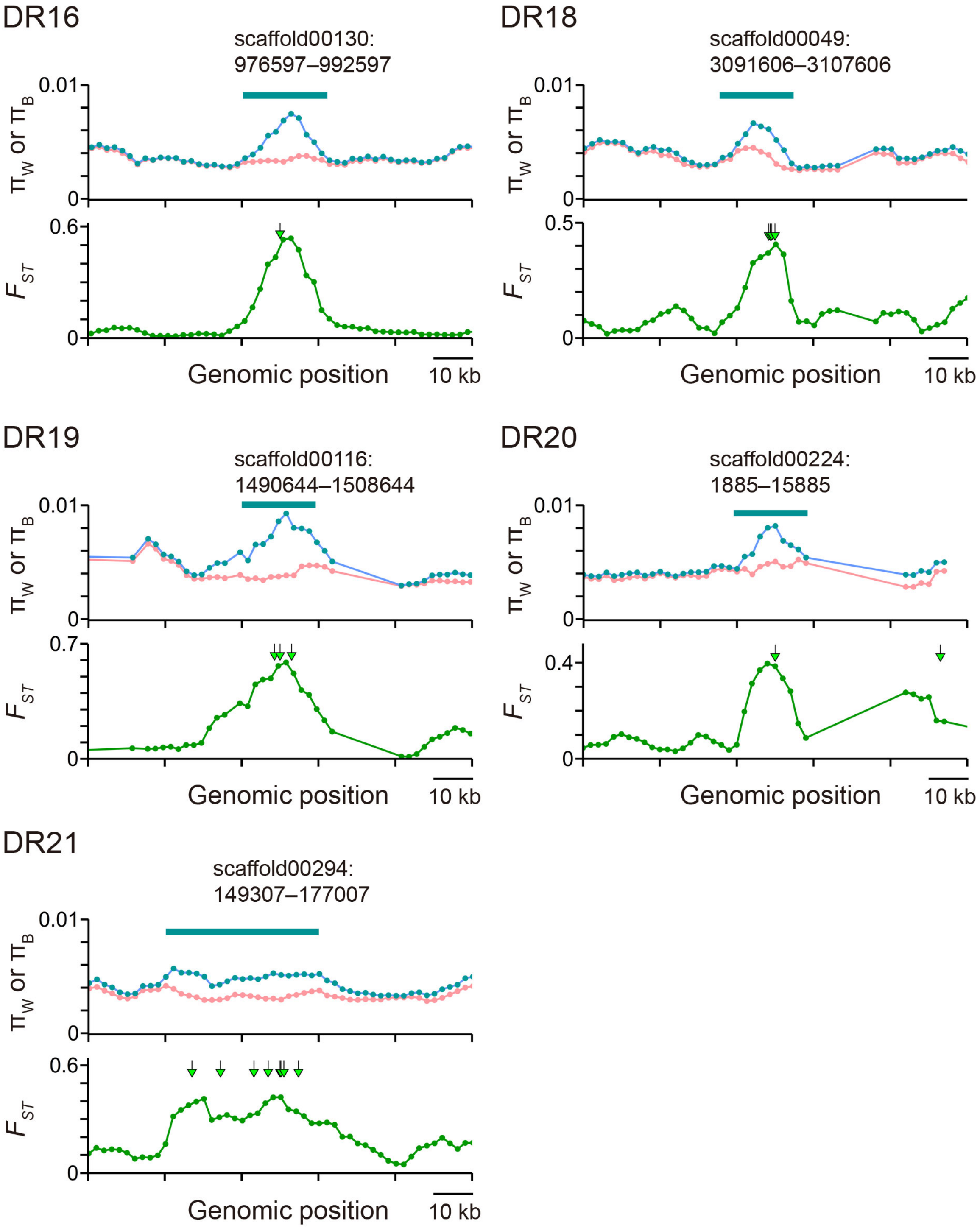

Figure S3. continued
