# Supplementary material for: Patterns of genomic differentiation between two Lake Victoria cichlid species, Haplochromis pyrrhocephalus and H. sp. ‘macula’
Source: BMC Evol Biol. 2019 Mar 4;19:68. doi: 10.1186/s12862-019-1387-2 (PMC6399900; doi:10.1186/s12862-019-1387-2)
Supplement: Supplementary file 5 — Figure S4. The origins of mutations in DRs. Three phylogenetic trees represent the accumulation of mutations in the common ancestral species of (A) Lake Victoria species, (B) Lake Victoria and riverine Haplochromis species, and (C) tribe Tropheini in Lake Tanganyika, Lakes Malawi, Victoria, and riverine Haplochromis species. The tree topologies constructed from sequences of each DR were consistent with (D) “Riverine origin” or (E) “Modern haplochromine origin.” Scale bars indicate the number of substitutions per site. (F) The LWS sequences were determined from three riverine species: H. sp. ‘katonga’ from Katonga, H. sp. ‘kitilda-rukwa’ from Kitilda-Rukwa, and H. sp. ‘muzu’ from Muzu. (PDF 554 kb) [file 12862_2019_1387_MOESM5_ESM.pdf]

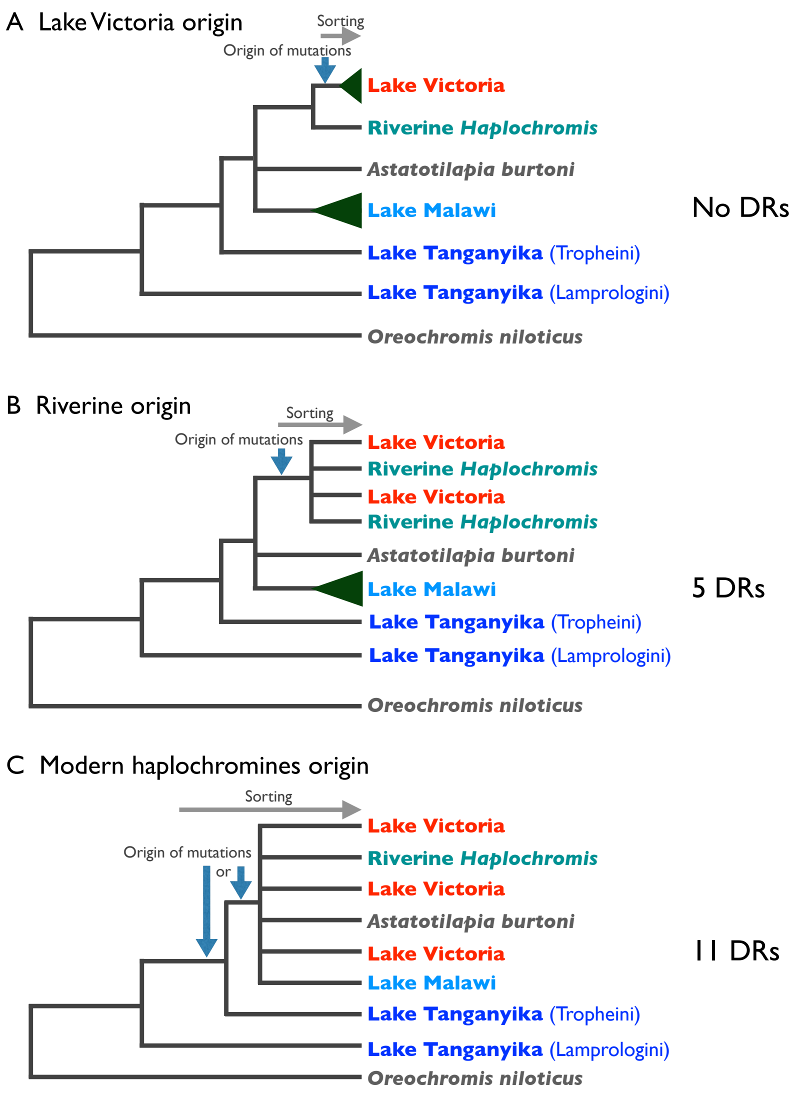

Figure S4. The origins of mutations in DRs. Three phylogenetic trees represent the accumulation of mutations in the common ancestral species of (**A**) Lake Victoria species, (**B**) Lake Victoria and riverine *Haplochromis* species, and (**C**) tribe Tropheini in Lake Tanganyika, Lakes Malawi, Victoria, and riverine *Haplochromis* species. The tree topologies constructed from sequences of each DR were consistent with (**D**) “Riverine origin” or (**E**) “Modern haplochromine origin.” Scale bars indicate the number of substitutions per site. (F) The LWS sequences were determined from three riverine species: *H.* sp. ‘katonga’ from Katonga, *H.* sp. ‘kitilda-rukwa’ from Kitilda-Rukwa, and *H.* sp. ‘muzu’ from Muzu.

D Riverine origin

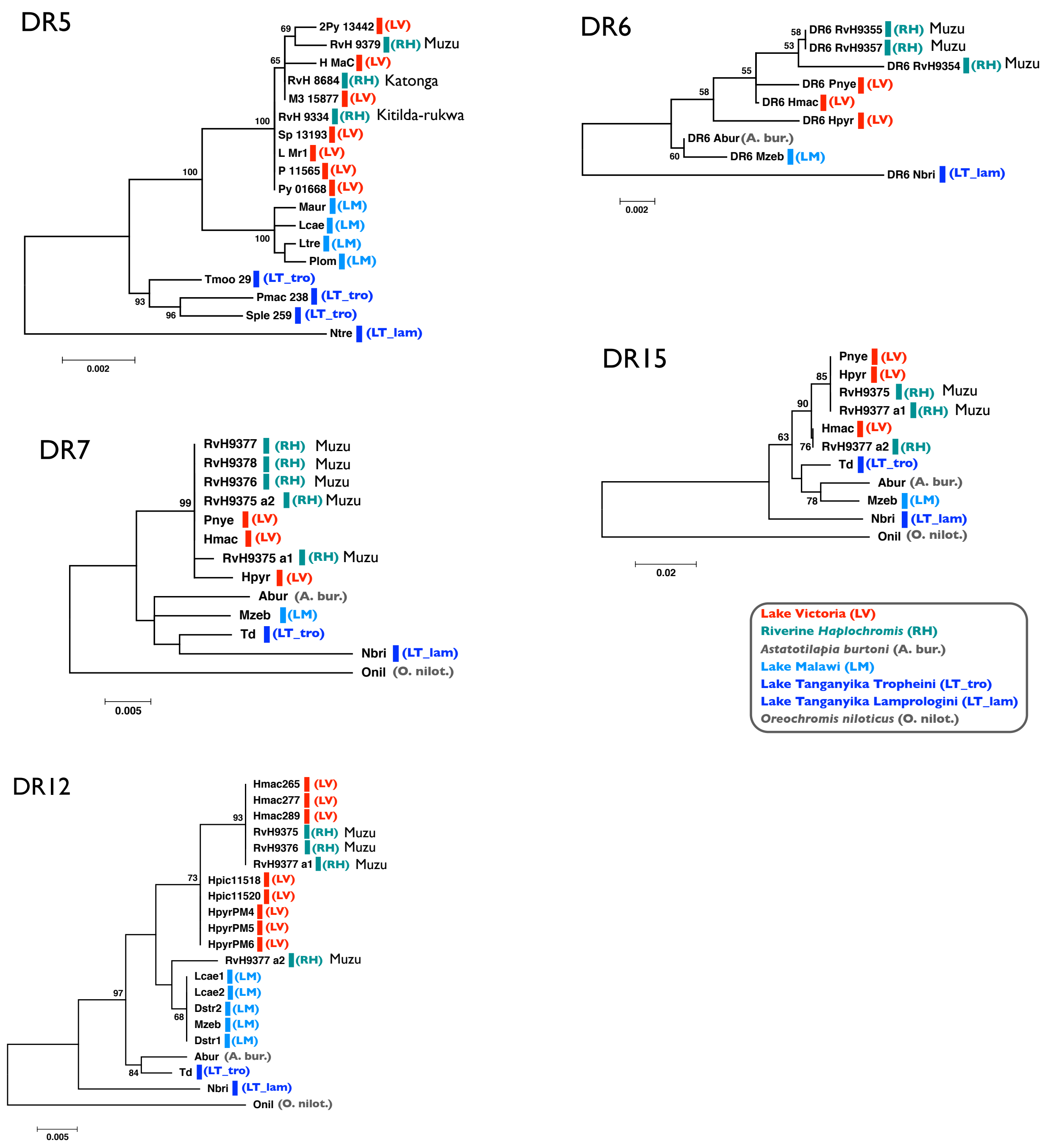

Figure S4. continued

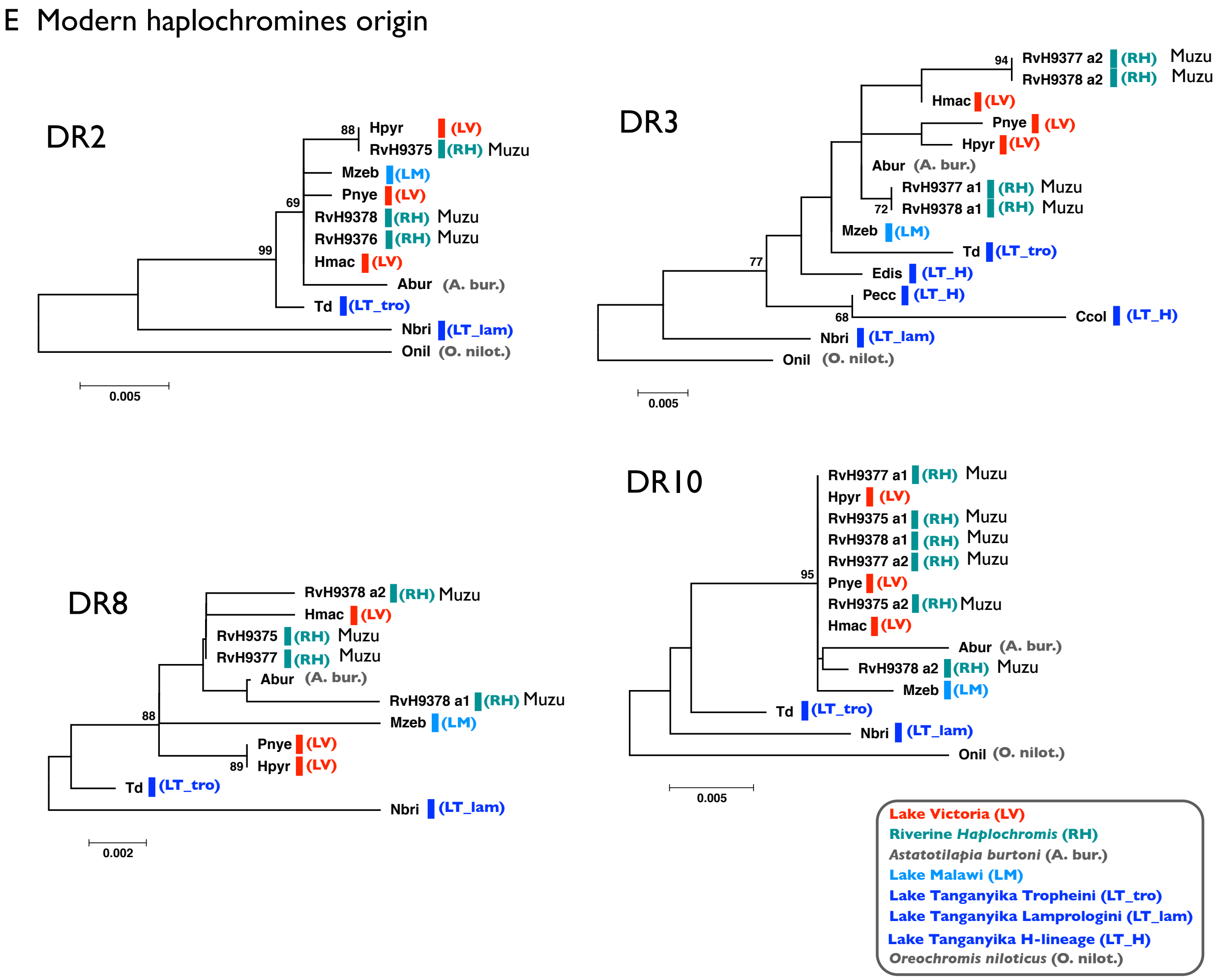

Figure S4. continued

E Modern haplochromines origin

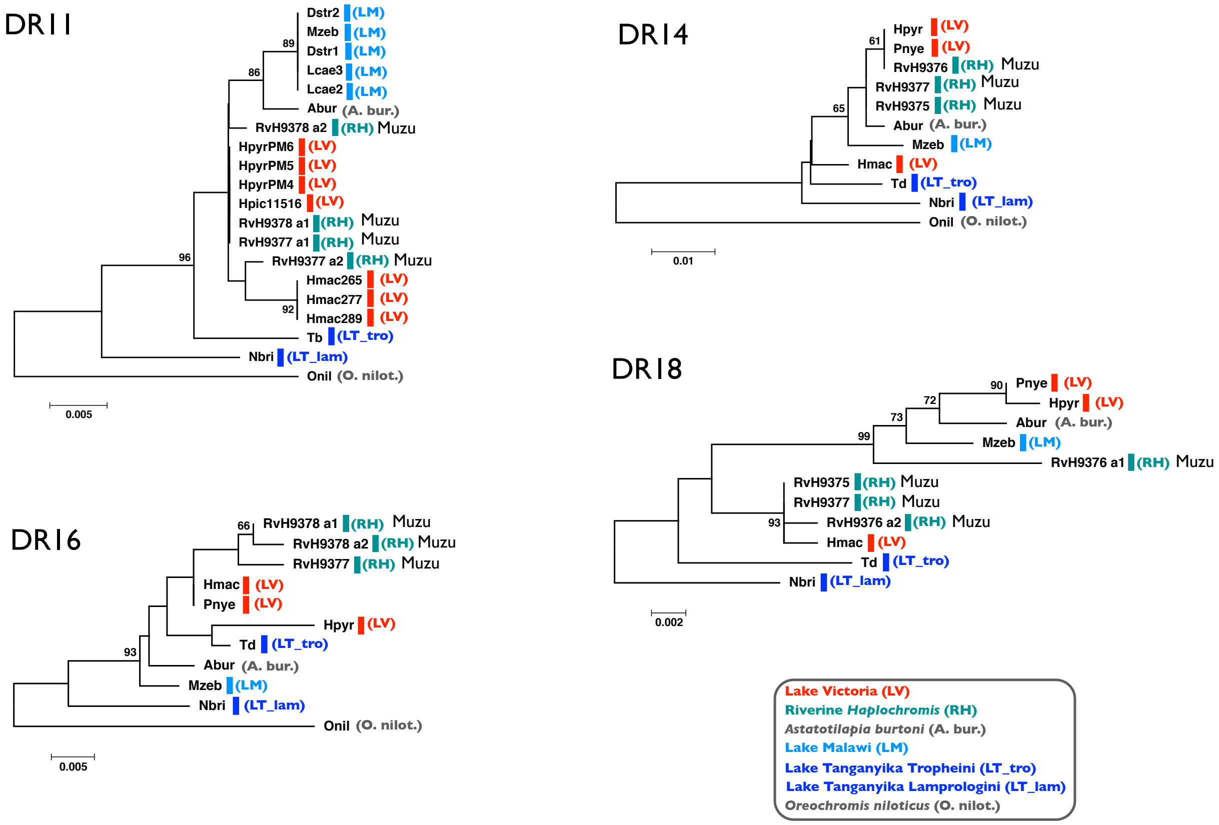

Figure S4. continued

E Modern haplochromines origin

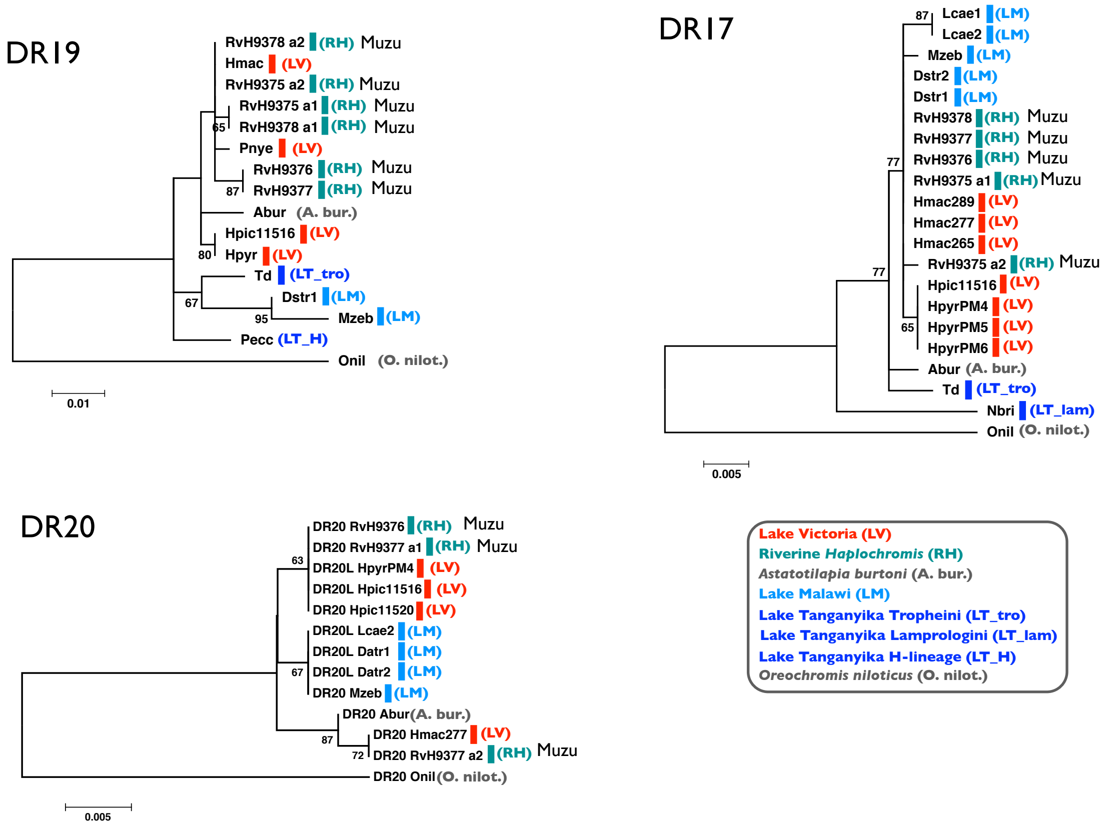

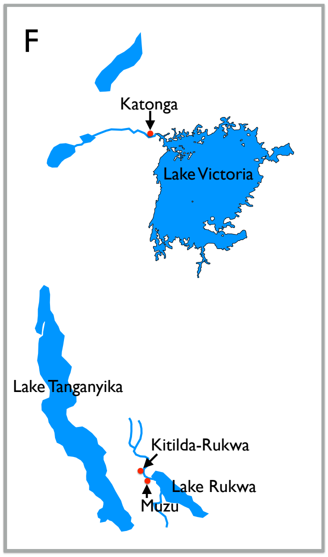

Figure S4. continued
