# Supplementary material for: Patterns of genomic differentiation between two Lake Victoria cichlid species, Haplochromis pyrrhocephalus and H. sp. ‘macula’
Source: BMC Evol Biol. 2019 Mar 4;19:68. doi: 10.1186/s12862-019-1387-2 (PMC6399900; doi:10.1186/s12862-019-1387-2)
Supplement: Supplementary file 6 — Table S1. Genes in DRs. (PDF 149 kb) [file 12862_2019_1387_MOESM6_ESM.pdf]

Table S1. Genes in DRs

DRs	Gene name	Sequence ID	Predicted roles in adaptation and speciation	References	Biological roles
DR1	diaphanous	XM_005724172	Developmental process, cell movement, auditory	[1]	Regulation of actin driven cellular processes
DR2	ventral anterior homeobox 2	XM_005751059	Development of retina	[2]	Major role in ventralizing embryonic retina
DR3	prostaglandin d2 receptor 2	XM_005455229	Unknown	[3]	Allergic responses
	G-protein coupled receptor 4 (GPR4)	XM_005750017	Adaptation to different oxygen concentrations	[4]	Regulation of breathing by CO_2 stimulation
	UDP-glucuronosyltransferase 2b15	XM_005750038	Unknown	[5]	Steroid metabolism
DR4	hemicentin-1	XM_005749930	Unknown	[6]	Maintain tissue and organ integrity
DR5	long wavelength-sensitive opsin	LWS CDS	Speciation by sensory drive	[7, 8]	Photoreceptor activity
DR6	netrin receptor UNC5c	XM_005735193	Brain development	[9]	Regulation of dorsal guidance of hindbrain axons
DR7	general transcription factor IIH subunit 1	XM_005730166	Unknown	[10]	Transcription and DNA repair
DR8	intestinal mucin	XM_005730137	Host-specific microbiota composition	[11]	Main component of intestinal mucus
DR9	hepatocyte growth factor receptor	XM_005751143	Morphogenesis of fin muscles affecting mobility	[12]	Growth of epithelial cells, migration of myogenic precursor cells
DR10	tbx3* (30 kp downstream of DR10)	XM_005720503	Developmental process	[13]	Various developmental process
DR11	ap-4 complex subunit epsilon	XM_005727983	Unknown	[14]	Vesicle trafficking
	cytochrome p450 aromatase type II	XM_005727984	Sexual differentiation of the brain and reproductive behavior	[15]	Estrogen metabolism
	gliomedin	XM_005727986	Unknown	[16]	Formation of nodes of Ranvier
DR12	melanopsin A	XM_005749719	Photic regulation of	[17]	Photoreceptor activity

			circadian clocks		
DR13	No gene	-	-	-	-
DR14	Hypothetical protein	XM_005732149	-	-	-
	No high similarity with known protein	XM_005732150	-	-	-
DR15	Uncharacterized ncRNA	XR_312020	-	-	-
DR16	aryl hydrocarbon receptor nuclear translocator	XM_005743315	Adaptation to different oxygen concentrations	[18]	Physiological adaptation to hypoxia
DR17	UDP-*N*-acetylglucosamine transporter	XM_004570512	Unknown	[19]	Nucleotide-sugar transporter, vertebral malformations
	U3 small nucleolar ribonucleoprotein protein imp3	XM_005720962	Unknown	[20]	Ribosomal RNA processing
DR18	peptidyl-prolyl cis-trans isomerase H	XM_005733013	Unknown	[21]	Post-translational modification
	transcription initiation factor TFIID subunit 10	XM_005733015	Early embryonic development	[22], [23]	Transcriptional activation
	G-protein coupled receptor 160	XM_005941175	Unknown	[24]	G-protein coupled receptor activity
DR19	type II cytoskeletal 5	XM_005742045	Epidermis development	[25], [26]	Cytoskeletal component
DR20	hydroperoxide isomerase aloxe3	XM_005748525	Epidermis development	[27]	Hepoxilin metabolism
	macrophage mannose receptor 1	XM_005951547	Unknown	[28]	Phagocytotic responses
DR21	Ras-related protein rab-11a	XM_005750946	Unknown	[29]	Vascular endothelial-cadherin recycling
	RNA-binding protein mex3a	XM_005750947	Brain aging	[30]	RNA-binding activity

*This DR did not contain a gene, but *tbx3* was located 30 kbp downstream of the DR.

References

1. Bogdan S, Schultz J, Grosshans J: **Formin'cellular structures: physiological roles of Diaphanous (Dia) in actin dynamics**. *Communicative & integrative biology* 2013, **6**(6):e27634.
2. Zhang Q, Eisenstat DD: **Roles of homeobox genes in retinal ganglion cell differentiation and axonal guidance**. *Adv Exp Med Biol* 2012, **723**:685-691.
3. Saito S, Tsuda H, Michimata T: **Prostaglandin D2 and reproduction**. *American Journal of Reproductive Immunology* 2002, **47**(5):295-302.
4. Kumar NN, Velic A, Soliz J, Shi Y, Li K, Wang S, Weaver JL, Sen J, Abbott SB, Lazarenko RM: **Regulation of breathing by CO2 requires the proton-activated receptor GPR4 in retrotrapezoid nucleus neurons**. *Science* 2015, **348**(6240):1255-1260.
5. Jenkinson C, Petroczi A, Naughton D: **Effects of dietary components on testosterone metabolism via UDP-glucuronosyltransferase (UGT)**. *Frontiers in endocrinology* 2013, **4**(80).
6. Xu X, Xu M, Zhou X, Jones OB, Moharomd E, Pan Y, Yan G, Anthony DD, Isaacs WB: **Specific structure and unique function define the hemicentin**. *Cell & bioscience* 2013, **3**(1):1.
7. Terai Y, Seehausen O, Sasaki T, Takahashi K, Mizoiri S, Sugawara T, Sato T, Watanabe M, Konijnendijk N, Mrosso HD *et al*: **Divergent selection on opsins drives incipient speciation in Lake Victoria cichlids**. *PLoS Biol* 2006, **4**(12):e433.
8. Seehausen O, Terai Y, Magalhaes IS, Carleton KL, Mrosso HD, Miyagi R, van der Sluijs I, Schneider MV, Maan ME, Tachida H *et al*: **Speciation through sensory drive in cichlid fish**. *Nature* 2008, **455**(7213):620-626.
9. Kim D, Ackerman SL: **The UNC5C netrin receptor regulates dorsal guidance of mouse hindbrain axons**. *The Journal of Neuroscience* 2011, **31**(6):2167-2179.
10. Egly J-M, Coin F: **A history of TFIIH: two decades of molecular biology on a pivotal transcription/repair factor**. *DNA repair* 2011, **10**(7):714-721.
11. Etzold S, Juge N: **Structural insights into bacterial recognition of intestinal mucins**. *Current opinion in structural biology* 2014, **28**:23-31.
12. Haines L, Neyt C, Gautier P, Keenan DG, Bryson-Richardson RJ, Hollway GE, Cole NJ, Currie PD: **Met and Hgf signaling controls hypaxial muscle and lateral line development in the zebrafish**. *Development* 2004, **131**(19):4857-4869.
13. Papaioannou VE: **The T-box gene family: emerging roles in development, stem cells and cancer**. *Development* 2014, **141**(20):3819-3833.
14. Hirst J, Irving C, Borner GH: **Adaptor protein complexes AP-4 and AP-5: new players in endosomal trafficking and progressive spastic paraplegia**. *Traffic* 2013, **14**(2):153-164.
15. Hemmer-Hansen J, Therkildsen NO, Meldrup D, Nielsen EE: **Conserving marine biodiversity: insights from life-history trait candidate genes in Atlantic cod (Gadus morhua)**. *Conservation Genetics* 2013, **15**(1):213-228.
16. Stathopoulos P, Alexopoulos H, Dalakas MC: **Autoimmune antigenic targets at the node of Ranvier in demyelinating disorders**. *Nat Rev Neurol* 2015, **11**(3):143-156.
17. Hankins MW, Peirson SN, Foster RG: **Melanopsin: an exciting photopigment**. *Trends Neurosci* 2008, **31**(1):27-36.
18. Wolff M, Jelkmann W, Dunst J, Depping R: **The Aryl Hydrocarbon Receptor Nuclear Translocator (ARNT/HIF-1beta) is influenced by hypoxia and hypoxia-mimetics**. *Cell Physiol Biochem* 2013, **32**(4):849-858.
19. Thomsen B, Horn P, Panitz F, Bendixen E, Petersen AH, Holm LE, Nielsen VH, Agerholm JS, Arnbjerg J, Bendixen C: **A missense mutation in the bovine SLC35A3 gene, encoding a UDP-N-acetylglucosamine transporter, causes complex vertebral malformation**. *Genome Res* 2006, **16**(1):97-105.
20. Phipps KR, Charette J, Baserga SJ: **The small subunit processome in ribosome biogenesis-progress and prospects**. *Wiley Interdiscip Rev RNA* 2011, **2**(1):1-21.
21. Reidt U, Wahl MC, Fasshauer D, Horowitz DS, Lührmann R, Ficner R: **Crystal Structure of a Complex Between Human Spliceosomal Cyclophilin H and a U4/U6 snRNP-60K Peptide**. *Journal of Molecular Biology* 2003, **331**(1):45-56.

22. Indra AK, Mohan WS, 2nd, Frontini M, Scheer E, Messaddeq N, Metzger D, Tora L: **TAF10 is required for the establishment of skin barrier function in foetal, but not in adult mouse epidermis**. *Dev Biol* 2005, **285**(1):28-37.
23. Goodrich JA, Tjian R: **Unexpected roles for core promoter recognition factors in cell-type-specific transcription and gene regulation**. *Nat Rev Genet* 2010, **11**(8):549-558.
24. Takeda S, Kadowaki S, Haga T, Takaesu H, Mitaku S: **Identification of G protein-coupled receptor genes from the human genome sequence**. *FEBS letters* 2002, **520**(1):97-101.
25. Gu LH, Coulombe PA: **Keratin function in skin epithelia: a broadening palette with surprising shades**. *Curr Opin Cell Biol* 2007, **19**(1):13-23.
26. Fischer B: **p53 and TAp63 promote terminal keratinocyte differentiation in breeding tubercles of the zebrafish**. Universität zu Köln; 2013.
27. Krieg P, Rosenberger S, de Juanes S, Latzko S, Hou J, Dick A, Kloz U, van der Hoeven F, Hausser I, Esposito I: **Aloxe3 knockout mice reveal a function of epidermal lipoxygenase-3 as hepoxilin synthase and its pivotal role in barrier formation**. *Journal of Investigative Dermatology* 2013, **133**(1):172-180.
28. Zheng F, Asim M, Lan J, Zhao L, Wei S, Chen N, Liu X, Zhou Y, Lin L: **Molecular cloning and functional characterization of mannose receptor in zebra fish (Danio rerio) during infection with aeromonas sobria**. *International journal of molecular sciences* 2015, **16**(5):10997-11012.
29. Yan Z, Wang Z-G, Segev N, Hu S, Minshall RD, Dull RO, Zhang M, Malik AB, Hu G: **Rab11a Mediates Vascular Endothelial-Cadherin Recycling and Controls Endothelial Barrier Function**. *Arteriosclerosis, thrombosis, and vascular biology* 2015:ATVBAHA. 115.306549.
30. Baumgart M, Groth M, Priebe S, Savino A, Testa G, Dix A, Ripa R, Spallotta F, Gaetano C, Ori M: **RNA‐seq of the aging brain in the short‐lived fish N. furzeri–conserved pathways and novel genes associated with neurogenesis**. *Aging cell* 2014, **13**(6):965-974.
